# Determination of the p*K*_a_ and Concentration of NMR-Invisible Molecules and Sites Using NMR Spectroscopy

**DOI:** 10.1021/acs.analchem.4c03596

**Published:** 2024-12-03

**Authors:** Haider Hussain, Yaroslav Z. Khimyak, Matthew Wallace

**Affiliations:** School of Chemistry, Pharmacy and Pharmacology, University of East Anglia, Norwich Research Park, Norwich NR4 7TJ, United Kingdom

## Abstract

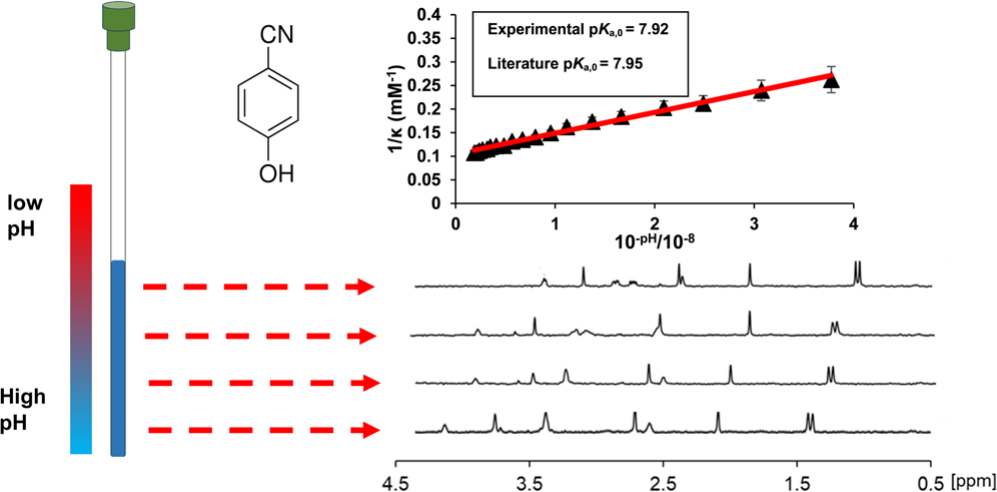

NMR spectroscopy is a very powerful tool for measuring the dissociation constants (p*K*_a_) of molecules, requiring smaller quantities of samples of lower purity relative to potentiometric or conductometric methods. However, current approaches are generally limited to those molecules possessing favorable pH-dependent NMR properties. Typically, a series of 1D experiments at varying pH are performed, and the p*K*_a_ is obtained by fitting the observed chemical shift of the analyte as a function of pH using nonlinear routines. However, the majority of polymers, biomolecules, and inorganic species do not present favorable NMR resonances. Either the resonances are not observable or too broad, or the unambiguous interpretation of the NMR data is impossible without resorting to complex 2D experiments due to spectral overlap. To overcome these fundamental limitations, we present a method to obtain the p*K*_a_ values and concentrations of acidic species without their direct observation by NMR. We instead determine the quantity of acidic protons removed from the species along a concentration gradient of an organic base in a single ^1^H chemical shift imaging experiment that can be run under automation. The p*K*_a_ values are determined via simple linear plots, avoiding complex and potentially unreliable nonlinear fitting routines.

The acid dissociation constant, *K*_a_, is a fundamental property which can be used to predict whether a molecule or ion will be protonated or deprotonated under different conditions.^1^*K*_a_, typically represented by its negative logarithm p*K*_a_, attracts significant interest in the areas of food science, pharmaceutical chemistry, and organic synthesis among other areas.^2^ In drug discovery, p*K*_a_ values are used to predict drug–target interactions and drug solubility.^3^ In materials science, the p*K*_a_ of polymers provides information about their self-assembly and complexation properties. For example, an understanding of the p*K*_a_ value of nucleic acid–polymer complexes is needed to prepare stable nucleic acids for therapeutic use.^4^ Additionally, inorganic ions such as phosphorus or nitrogen species play an important role in biological systems while proteins exhibit a pH-dependent charge which determines their solubility, stability, and separation properties.^5^

NMR spectroscopy is a valuable technique to measure p*K*_a_ as it offers the advantages of studying analytes using small volumes with equipment that is available in most research institutions.^6^ Furthermore, we demonstrated how, when the analyte can be observed by ^1^H NMR, p*K*_a_ can be determined using a combination of pH gradients and 2D chemical shift imaging (CSI).^7^ This allows for a “single shot” determination of p*K*_a_ in one NMR experiment, saving material and time.^7−9^ However, a restriction on most NMR methods is that they require the analyte to display chemical shifts which can be clearly observed to change as a function of pH, or else require the concentration of analyte to be known.^10^ However, not all systems of interest can be characterized in this manner. A striking case is polymeric systems with molecular weights above 20,000 g/mol that typically display very broad resonances.^11^ Additionally, even for systems that have a lower molecular weight, given enough complexity, the spectral overlap will make the resolution of separate resonances impractical. The ChEMBL database displays 1,913,280 small molecules that are preclinical, of which approximately 20% have a molecular weight over 600 g/mol and are therefore likely to exhibit overalpping resonances on a standard ^1^H NMR spectrum.^12^ Moreover, the BMRB (Biological Magnetic Resonance) databank contains more than 15,000 entries regarding peptides and proteins that can be investigated using solution-state NMR, but for which 2D NMR experiments and isotopic labeling would be needed in many cases to assess their pH-dependent behavior.^13^ Such “NMR restricted” molecules and ions are of great interest in a variety of fields and would otherwise benefit from p*K*_a_ determination by NMR.

Here, we present a more general NMR method that has the capacity to encompass these restricted molecules, expanding the range of molecules that can be probed by NMR, hence providing avenues for exploring their p*K*_a_-associated activity that was not previously possible while also enabling the analysis of species whose toxicity, volatility, or limited availability may preclude the use of conventional potentiometric titrations. The new method determines the p*K*_a_ of molecules with no or poorly observable NMR resonances by measuring the quantity of protons transferred to a basic indicator along pH gradients in 5 mm NMR tubes. We demonstrate the measurement of the p*K*_a_ values of a range of small organic and inorganic molecules in excellent agreement with the literature values. We apply our method on complex systems to determine the effective p*K*_a_ of poly(acrylic acid) and estimate the isoelectronic point, p*I*, of wheat germ agglutinin.

## Experimental Section

### Materials

All chemicals were purchased from Fisher Scientific or Sigma-Aldrich and used as received. Stock H_2_O (18.2 MΩ.cm) solution of the NMR indicators (Table 1) were prepared and used throughout the study. Indicators used were 2-methylimidazole (2-MI), 1,2,4-triazole, formate, acetate, and 2,6-lutidine. Formate, acetate, and were included with sodium as their cation. The analytes studied were boric acid, 4-cyanophenol (4-CN), phosphoric acid (H_3_PO_4_), sodium dihydrogen phosphate (NaH_2_PO_4_), glycine hydrochloride, hydroxylammonium chloride (NH_3_OHCl), ammonium chloride (NH_4_Cl), benzoic acid, and glycolic acid. Stock solutions of the analytes in H_2_O were prepared at 10 mM concentrations of each except phosphoric acid, which was prepared at 60 mM to explore the scope of the methodology. Polyacrylic acid (PAA, Mw = 240 kDa) and lectin from *Triticum vulgaris* (also known as wheat germ agglutinin (WGA)) were used as model analytes for measuring effective p*K*_a_ of polymers and p*I* of proteins, respectively. The stock solution contained 0.2 mM of 2,2-dimethyl-2-silapentane-5-sulfonate (DSS) which acted as a chemical shift reference and 0.01% of either DMSO or 1,4-dioxane which acted as an integral reference (choice was based on which reference compound had least spectral overlap with the analyte). The correction factors of dioxane and DMSO were determined by performing a 2D CSI experiment in a solution with a known concentration of base and a standard concentration of the integral references (Section S6).

**Table 1:** Basic NMR pH Indicators Used in This Work

Indicator	p*K*_a,0_	δ_H_/ppm	δ_L_/ppm	pH range^a^
2-MI	7.96	7.270	6.958	9–7
2,6-lutidine	6.75	2.707	2.456	8–6
Acetate	4.76	2.083	1.906	5.5–3.5
Formate	3.75	8.266	8.441	4.5–2.5
1,2,4-triazole	2.45	9.193	8.352	3–1

a The pH range accessible when measuring the chemical shift of the indicator. The uncertainty in the measurement of chemical shift precludes accessing pH values beyond this range (Section S3).

**Figure 1:**
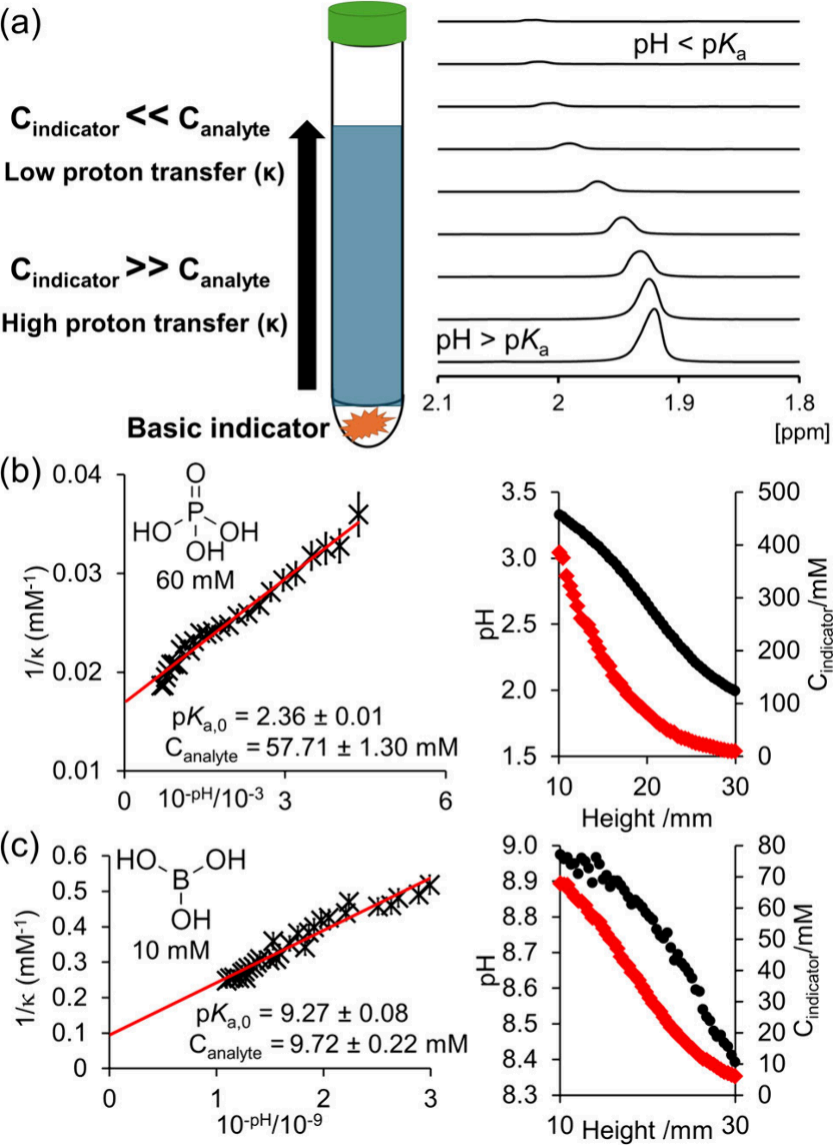
(a) A concentration gradient of a basic indicator is established in an NMR tube, allowing measurement of the quantity of protons transferred from acid to base as a function of pH. (b) Plot of 1/κ versus 10^–pH^ for 60 mM H_3_PO_4_ (left) and plot of pH (black circle) and C_indicator_ (red diamond) versus height from tube base (right). (c) Plot of 1/κ versus 10^–pH^ for 10 mM boric acid (left) and plot of pH and C_indicator_ versus height from tube base (right).

Limiting chemical shifts for the indicators (Table 1) were obtained by measuring the chemical shift of the indicator in a homogeneous solution containing HCl (0.01 M) with a concentration gradient of the basic indicator (Section S7) with the exception of the limiting chemical shifts of 2,6-lutidine and which were obtained from our previous work.^7^ κ was assumed equal to the concentration of HCl as it is a strong acid. All experiments were performed in 5 mm Wilmad 528-PP NMR tubes. To establish a pH gradient using bases that were solid at room temperature, 4–5 mg of solid base was weighed into the tube, and four 2 mm diameter glass beads were placed on top of the base (Figure 1a). An aliquot of the analyte solution was drawn up in a 9″ Pasteur pipet and gently layered on top of the glass beads to a height of 40–50 mm from the base of the NMR tube. However, when 2,6-lutidine (which is liquid at room temperature) was used as a diffusant, an aqueous solution (2 M, 30 μL) was pipetted on top of a 500 μL analyte solution where it floated due to its lower density. The NMR tubes were stored in the sample changer rack at 21–22 °C until the optimum time for running NMR experiments. Experiments were run within 20 min of the optimum time, t_op_, at which the gradient was predicted to have developed (Section S5).^7^

### NMR

Experiments were performed at 298 ± 0.5 K on a Bruker Avance III 500 MHz spectrometer operating at 500.21 MHz for ^1^H. CSI experiments were performed using a gradient phase encoding sequence based on that of Trigo-Mouriño et al.^14^ but incorporating double echo excitation sculpting for water suppression (Bruker library zgesgp), with the ramped gradient pulse included at the end of the water suppression block. A balancing delay to compensate for this gradient pulse and recovery delay (200 μs) were added to the last echo of the water suppression block, while a spoil gradient pulse (1 ms, 25 G/cm) was inserted at the end of the relaxation period (2.0 s) to destroy any remaining transverse magnetization (Section S14). The signal acquisition time was 2.04 s with a sweep width of 16 ppm. The encoding gradient pulse (172 μs, smoothed square) was varied between −18.8 and 18.8 G/cm in 64 steps. Here, 4 ms Gaussian 180° pulses were employed for water suppression, while the hard 90° pulse was 10 μs. Four scans were acquired at each step, while 16 dummy scans were acquired prior to signal acquisition, giving a total acquisition time of 20 min. The vertical range of the experiment (cnst0, Section S14) was set to 2.6 cm, and the spatial resolution can thus be assumed equal to 0.41 mm, such that each 1D spectrum in the data set arises from a 0.41 mm thick slice. All spectra were referenced to DSS (0 ppm). NMR data were processed using Bruker TopSpin 3.6.5. C_indicator_ and the pH of each row of the CSI data set were determined experimentally from the integrals and chemical shifts of the indicator resonances, respectively, on each spectrum. C_analyte_ was determined by linear fitting, as described below. Scripts for the acquisition and processing of NMR data are provided in Section S15–17, while a spreadsheet is supplied as additional Supporting Information.

## Results and Discussion

The pH at each position along the sample is determined from the chemical shift of an indicator molecule, δ_obs_, by eq 1:^7^
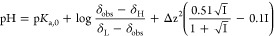
1where δ_H_ and δ_L_ are the limiting chemical shifts of the protonated and deprotonated species respectively, Δz^2^ is the difference in the square of the charge of the indicator between the protonated and deprotonated states (−1 and +1 for non-nitrogenous and nitrogenous species in our work, respectively), and I is the ionic strength (Section S2).^15−17^ Basic indicators used in this work are provided in Table 1; p*K*_a,0_ values were obtained from literature sources.^7,18,19^

Assuming protonation of the indicators from H_2_O is negligible due to their low basicity (Section S11), the concentration of protons transferred from the acidic analyte, κ, is formulated as
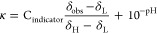
2
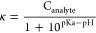
3where C_indicator_ is the concentration of basic indicator which varies across the NMR tube and is measured at each position across the sample through the use of an integral reference (see Section S6), and C_analyte_ is the total concentration of acidic analyte. C_analyte_ is taken to be homogeneous across the sample and assumed to be the concentration of analyte in the solution. The second term on the right of eq 2 is significant when studying compounds with relatively low p*K*_a_ values such as phosphoric acid (Section S12). Equation 3 can be expressed in linear form as eq 4 (Section S1):
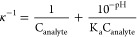
4

**Figure 2:**
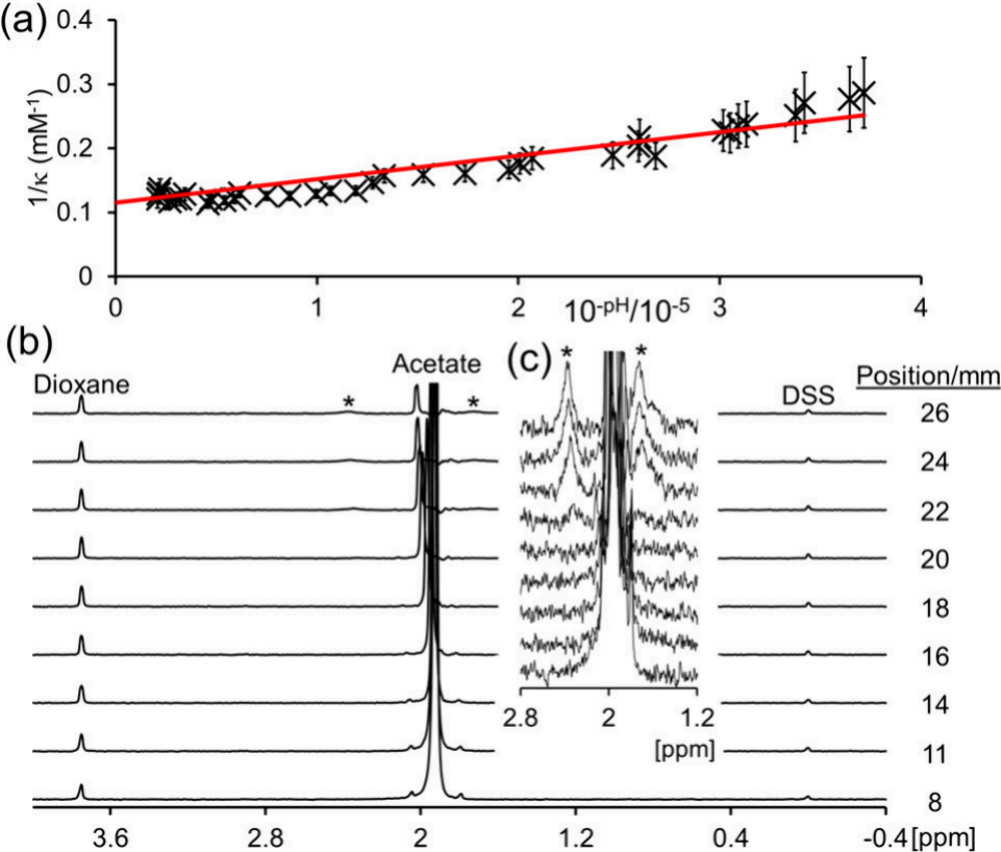
(a) Plot of 1/κ versus 10^–pH^ for poly(acrylic acid) (10 mM COOH groups) with sodium acetate as indicator. (b) Partial ^1^H NMR spectra at different positions from the base of the NMR tube. (c) Expansion of the acetate and PAA region is presented to highlight the disappearance of the PAA resonances (∗).

κ is measured using eq 3, and the pH is measured using eq 2. Equation 4 forms a linear plot of κ^–1^ versus 10^–pH^ with a gradient of 1/K_a_C_analyte_ and intercept of 1/C_analyte_ where K_a_ is of the “mixed” type (the dissociation constant is written in terms of the concentration of the conjugate acid/base and activity of H^+^, Section S1).^20^Figure 1 showcases the scheme used in the experiment and the p*K*_a_ and C_analyte_ obtained for boric acid and phosphoric acid. We note that p*K*_a_ and C_analyte_ can also be obtained by nonlinear fitting of eq 3 (Section S10).

p*K*_a,0_ and C_analyte_ were determined from the gradient and intercept of a plot of 1/κ versus 10^–pH^ (Figure 1). The values of p*K*_a_ obtained by linear fitting are within 0.3 units of the literature values (uncertainty values obtained are the 95% confidence intervals of the least-squares regression line) across a wide range of analytes (Table 2).^21−32^ The indicators used were chosen to be within 2 units of the expected p*K*_a_ of the analyte and give pH ranges that cover ±1 units across the p*K*_a_ of the indicator. Obtaining data at pH values more than one unit higher or lower than the p*K*_a_ of the indicator introduces a high degree of uncertainy (Section S3). All analytes studied with their respective parameters and fitted values of p*K*_a_ and C_analyte_ are listed in Table 2. p*K*_a_ measurements obtained were comparable in accuracy to standard methods.^33−36^ To highlight the potential of our method to determine the acidities of polymeric systems, we determined a p*K*_a_ of poly(acrylic acid) (Mw 240 kDa, 10 mM COOH groups) with sodium acetate as an indicator. Given that PAA has multiple acidic sites, the p*K*_a_ is a function of the degree of ionization of the polymer;^37^ hence, a plot of κ^–1^ versus 10^–pH^ deviates slightly from linearity (Figure 2a). Our method returns a single effective p*K*_a_ of all sites of 4.83 ± 0.02 with an R^2^ of 0.94, in good agreement with the literature value of 4.5 reported when the degree of ionization approaches zero, while the total concentration of acidic groups (9.65 mM ± 1.50) is also recovered by our fitting (Figure 2a).^37^

**Table 2:** Literature and Fitted Values of p*K*_a_ and C_analyte_

Compound	Fitted p*K*_a,0_	Literature p*K*_a,0_	C_analyte_ /mM^a^	Indicator
Glycine.HCl	2.63 ± 0.23	2.35^32^	9.28 ± 1.50	1,2,4-triazole
H_3_PO_4_^b^	2.36 ± 0.01	2.16^22^	57.71 ± 1.30	1,2,4-triazole
NaH_2_PO_4_^b^	7.36 ± 0.24	7.21^29^	8.95 ± 4.10	2-MI
4-CN	7.92 ± 0.03	7.95^30^	10.60 ± 0.25	2-MI
Boric acid^b^	9.27 ± 0.08	9.19^24^	9.72 ± 0.22	2-MI
NH_3_OHCl^b^	5.76 ± 0.23	5.94^22^	8.92 ± 1.50	2,6-lutidine
NH_4_Cl^b^	9.43 ± 0.27	9.24^26^	9.80 ± 3.40	2-MI
Benzoic acid	4.29 ± 0.04	4.20^31^	9.60 ± 0.24	Acetate
Glycolic acid	3.89 ± 0.06	3.89^27^	11.73 ± 1.02	Formate

a Concentrations were 10 mM, except H_3_PO_4_ (60 mM).

b Compounds do not exhibit distinct ^1^H resonances in H_2_O due to fast exchange of NH and OH protons.

The results on PAA highlight the potential of our technique for the determination of the effective p*K*_a_ of ^1^H inactive polymers which typically are hard to probe using NMR given their short T_2_ values and considerable peak broadening effects (Section S9).^38^ The resonances of PAA disappear as the concentration of sodium acetate increases along a concentration gradient (Figure 2b, c). Deprotonation of PAA will increase the hydrodynamic radius^37^ which is expected to cause a net migration of the polymer toward the diffusing base, in analogy to the diffusion of polygalacturonate toward diffusing calcium.^39^ The higher concentration and partial protonation of the PAA may lead to entanglement of the chains, causing broadening of the PAA resonances beyond detection. We note that the resonances of PAA also decrease up a concentration gradient of NH_3_, while no loss of signal is observed along a gradient of NaCl or in homogeneous solutions of PAA and sodium acetate (Section S9). These results suggest that the loss of the PAA resonances is related to the presence of a concentration gradient of base rather than deprotonation alone or diffusiophoresis.^40^ Our method does not depend on the direct observation of the chemical shift of PAA and, as a result, is advantageous in situations where the analyte agglomerates or diffuses in solution after deprotonation has taken place. As we are studying the reaction of acetate with protonated PAA toward the top of the NMR-active region of the sample, our method still returns accurate values of C_analyte_ and an effective p*K*_a_.

**Figure 3:**
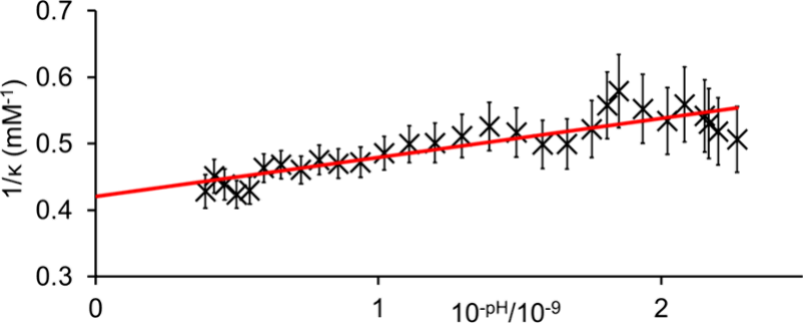
Plot of 1/κ versus 10^–pH^ for WGA with 2-methylimidazole as an indicator.

To highlight the potential of our method for studying the pH-dependent properties of proteins, simplifying the process and time needed to study these properties using solution state NMR, we used our method to estimate the isoelectric point of wheat germ agglutinin (WGA, 3.8 mg/mL) using 2-MI as the indicator (Figure 3). A p*I* value of 7.76 ± 0.4 was obtained, in good agreement with the observation of Rice and Etzler that agglutinins extracted from commercial wheat germ focused within the pH range 7–9.^41^ We note that the ionization state of the whole protein is dependent on the acidity and basicity of all titratable groups. Our method thus provides an effective p*K*_a_ of the whole molecule without the 2D NMR experiments and isotopic labeling required to determine the p*K*_a_ of individual titratable groups.^42^ C_analyte_ was calculated as 2.38 ± 0.24 mM. Assuming a molecular weight of 21 kDa, the concentration of protein is 180 μM which would suggest 13 ± 1 acidic sites per protein. This value is consistent with the ca. 10 acidic residues (glutamic acid or aspartic acid) reported in the sequences of WGA isolectins.^43^

## Conclusion

We have shown how the p*K*_a_ of any substance can be determined in a single NMR experiment at a concentration of acidic sites as low as 2 mM. With a standard NMR sample volume of *ca*. 500 μL, our approach provides a substantial saving in sample quantity and experimental time relative to conventional workflows based on 2D heteronuclear NMR experiments and manual adjustment of the sample pH. Furthermore, by avoiding completely the requirement for direct observation of the molecule of interest, we can analyze any molecule or ion including those that do not exhibit observable NMR resonances (NH_3_OH^+^), or else resonances that are too broad (PAA) or not pH responsive (WGA) for analysis based on the pH-dependence of their ^1^H chemical shifts. Knowledge of p*K*_a_ of these large systems could be used to inform the design of polymer–drug conjugates or liposomal formulations.^44^ Our approach could potentially be extended to study the degree of proton transfer between acidic and basic partners at different volume fractions of organic solvent, greatly accelerating the determination of the p*K*_a_ of compounds with insufficient solubility for direct analysis in water.^45−47^

## Data Availability

The data underlying this study are openly available at https://research-portal.uea.ac.uk/en/datasets/

## Supporting Information Available

Spreadsheet for analysis of data set (XLSX)
Derivation of eq 4; determination of pH, ionic strength and spatial position; estimation of uncertainty; estimation of uncertainty in κ; determination of optimum time; integral correction factors; determination of limiting chemical shifts; spectra of NaH_2_PO_4_; spectra of PAA; impact of protonation by H_2_O; plot of 1/k for all analytes; pulse sequence; and automation scripts for data processing (PDF)
