# Supplementary material for: Determination of the pKa and Concentration of NMR-Invisible Molecules and Sites Using NMR Spectroscopy
Source: Anal Chem. 2024 Dec 3;96(50):19858–62. doi: 10.1021/acs.analchem.4c03596 (PMC11656416; doi:10.1021/acs.analchem.4c03596)
Supplement: Supplementary file 2 — ac4c03596_si_002.pdf [file ac4c03596_si_002.pdf]

# Supporting information

# Determination of the p$K_a$ and concentration of NMR-invisible molecules and sites using NMR spectroscopy

Haider Hussain*, Yaroslav Z. Khimyak and Matthew Wallace*

School of Chemistry, Pharmacy and Pharmacology, University of East Anglia, Norwich Research Park, Norwich, NR4 7TJ, UK

*Corresponding Authors

Matthew Wallace: matthew.wallace@uea.ac.uk

Haider Hussain: haider.hussain@uea.ac.uk

S1. Derivation of Equation 4 .......... 2

S2. Determination of Ionic strength and spatial location of NMR spectra .......... 3

S3. Uncertainty in pH determined by NMR .......... 4

S4. Estimation of uncertainty in the determination of κ .......... 6

S5. Optimum time determination .......... 7

S6. Integral correction factors .......... 8

S7. Determining limiting chemical shifts of indicators .......... 9

S8. 2D $^1$H NMR chemical shift spectra array of sodium dihydrogen phosphate .......... 10

S9. $^1$H NMR spectra of homogenous polyacrylic acid with sodium acetate, and concentration gradients of $NH_3$ and NaCl .......... 11

S10. Alternative method for determining p$K_a$ and concentration of analyte .......... 13

S11. Impact of $H_2O$ on protonation of indicators .......... 14

S12. Determining the protons transferred to $H_2O$ for low p$K_a$ determination .......... 15

S13. Plot of 1/κ versus pH for all analytes .......... 16

S14. 2D pulse sequence for CSI (Bruker) .......... 22

S15. AU program to process raw CSI datasets .......... 23

S16. AU program to pick peak a chemical shift in CSI .......... 24

S17. AU program to integrate a peak in CSI .......... 25

## S1. Derivation of Equation 4

κ is defined as the molar concentration of protons transferred from the acidic analyte to the basic indicator. Conversely it is defined as the molar concentration of protons the base obtains from the acid. Hence, κ can be described by equation S1 and S2:

$$\kappa = C_{indicator}\, f_{H\,indicator} \tag{S1}$$

$$\kappa = C_{analyte}\, f_{L\,analyte} \tag{S2}$$

Where $C_{indicator}$ is the total concentration of basic indicator, $f_{H\,indicator}$ is the fraction of it protonated, $C_{analyte}$ is the total concentration of acidic analyte and $f_{L\,analyte}$ is the fraction of it deprotonated. To obtain $f_{H\,indicator}$ we use equation S3:

$$\delta_{obs} = \delta_L f_{L\ indicator} + \delta_H f_{H\,indicator} \tag{S3}$$

Equation S3 describes how the chemical shift of observed molecule is the average of the chemical shifts of the protonated and deprotonated states. Equation S1 and S3 are combined to give equation 2:

$$\kappa = C_{indicator}\, \frac{\delta_{obs} - \delta_L}{\delta_H - \delta_L} \tag{2}$$

This allows for determination of κ given knowledge of $\delta_L$ and $\delta_H$ of indicator (all respective indicator values used in this work are found in table 1). $f_{L\,analyte}$ can be obtained by manipulating the equilibrium dissociation constant $K_a$ to obtain equation S4:

$$K_a = \frac{[H^+] f_{L\,analyte}}{1 - f_{L\,analyte}} \tag{S4}$$

consequently equation S2 gets reformulated to obtain:

$$\kappa = \frac{C_{analyte}}{1 + 10^{pKa - pH}} \tag{3}$$

Rearranging 3 by taking the inverse of κ gives equation 4:

$$\kappa^{-1} = \frac{1}{C_{analyte}} + \frac{10^{-pH}}{K_a C_{analyte}} \quad (4)$$

$K_a$ obtained from equation 4 would be the apparent $K_a$. The thermodynamic $K_a$ is the $K_a$ at infinite dilution with respect to ionic strength. This is represented via the thermodynamic association constant equation:

$$K_{a,0} = \frac{\gamma_{H+}[H^+]\gamma_{A-}[A^-]}{\gamma_{HA}[HA]}$$

Where γ is the activity coefficient relating apparent $K_a$ to ideal $K_{a,0.}$ thermodynamic $K_a$ is obtained by determining the activity coefficients of $H^+$ and $A^-$ ( $\gamma_{HA}$ is assumed to equal 1 for analytes which are neutral in their protonated form and $\gamma_{A-}$ is assumed to equal 1 for analytes which are positive in their protonated form). Activity coefficients are calculated via the Davies equation:

$$\gamma = 10^{-0.51z^2(\frac{\sqrt{I}}{1+\sqrt{I}}-0.2I)} \quad (S5)$$

Where z is the charge of the ion and I is the ionic strength (See section S2 for how to calculate ionic strength). $\gamma_{H+}$ and $\gamma_{A-}$ are determined as the average of the activity coefficients of all the nmr slices.

## S2. Determination of Ionic strength and spatial location of NMR spectra

For accurate calculation of pH in this work, we explicitly calculate the ionic strength at each point along the pH gradient based on the concentration and charge of the indicator and ionic strength of the solution.

Ionic strength is calculated using the standard ionic strength equation:[50]

$$I = 0.5 \sum C_i {z_i}^2 \quad (S6)$$

Where C is concentration in mol/L and z is charge. This is done for each slice of the CSI experiment. Both the background ionic strength of the analyte solution, comprising the DSS and other potential buffers or indicators added to the solution (in present work for simplicity no buffers or other indicators were added) and the ionic strength contributed by the base indicator is calculated using equation S7:

$$I = I_0 + I_{base} + I_{analyte} \quad (S7)$$

Where $I_0$ is background ionic strength and $I_{base}$ is ionic strength contribution from base indicator and $I_{analyte}$ is ionic strength contribution from acidic analyte. Concentration of base indicator is calculated by measuring the integral of one of its peaks and relating it to a reference integral (see Section S6) then determining what fraction, f, of that concentration contributes to ionic strength (fraction protonated for nitrogenous bases and fraction

deprotonated for non-nitrogenous bases). f is estimated using a modified Henderson–Hasselbalch equation:

$$\mathrm{pH} = \mathrm{pK_{a,0}} + \log_{10}\left(\frac{f_{L\ indicator}}{f_{H\ indicator}}\right) = \mathrm{pK_{a,0}} + \log_{10}\left(\frac{f_{L\ indicator}}{1 - f_{L\ indicator}}\right) \quad \text{(S8)}$$

Obtaining $f_{L\ indicator}$ or $f_{H\ indicator}$ and multiplying them by the total concentration of the indicator at that slice gives a reasonable approximation of the concentration of non-nitrogenous base and nitrogenous base that contributes to ionic strength respectively. $I_{analyte}$ is determined to be the concentration of ionically active analyte species which is approximately equivalent to the apparent proton transferred for a non-nitrogenous analyte or the difference between the concentration of the acid and protons transferred if it is a nitrogenous analyte.

p$K_{a,0}$ in table 2 was obtained from the fitted $K_a$ (Equation 4), with $\gamma$ taken as the average value of $\gamma$ calculated for each useable row of the CSI dataset using Equation S5.

The spatial position of each row of the CSI dataset is calculated via equation S9:

$$p = \left(\varepsilon - \frac{E}{2} - 0.5\right) \times \frac{Z\text{-}range}{E} \quad \text{(S9)}$$

Where p is position of the experiment as measured from the centre of the NMR active window, ε is the slice number, E is the total number of experimental slices (64 in our experiments) and Z-range is window of NMR active region of the NMR tube in mm (26 mm). This allows for correlating the pH measurements to specific locations along the NMR tube.

## S3. Uncertainty in pH determined by NMR

Chemical shift measurements have some degree of uncertainty due to factors such as slight magnetic field inhomogeneity or temperature fluctuations amongst other factors. We performed CSI experiments on a homogenous solution of DSS and DMSO and observed the chemical shift uncertainty as 0.0007 ppm by calculating the standard deviation of the chemical shift of DMSO (referenced to DSS) along the 64 datasets of the homogenous solution. We assume the uncertainty to be 0.0007 ppm in this paper. We also simulated a pH gradient of sodium acetate and done uncertainty analysis on pH measurement with respect to δobs of sodium acetate (figure 1) and observed that uncertainty increases significantly once pH is ± 2 units of the p$K_a$ value of the indicator thus providing a clear window of when the indicator's pH measurement is reliable.

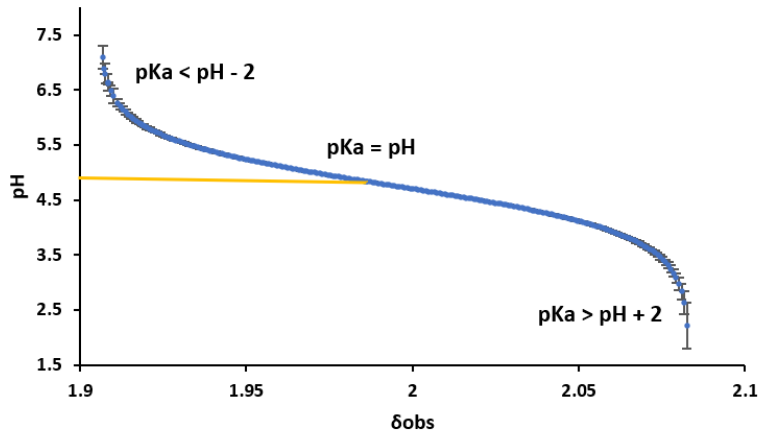

**Figure S1.** plot of pH calculated using equation 2 versus $^{1}H$ $\delta_{obs}$ of acetate (p$K_{a,0}$ = 4.76), yellow line crosses curve at p$K_{a,0}$ of acetate.

**Table S1.** $\delta_{obs}$ and pH values calculated from the [1]H chemical shift of sodium acetate and the comparison of pH difference when pH ≈ p$K_a$ of acetate (4.756) and when the pH is two units higher.

| δobs/ppm | pH | δobs*/ppm | pH* | ΔpH |
|---|---|---|---|---|
| 1.9790 | 4.90971 | 1.9797 | 4.902633 | 0.007078 |
| 1.9073 | 6.886828 | 1.9080 | 6.698008 | 0.188820 |

* δobs + 0.0007 ppm and the resultant change in pH

## S4. Estimation of uncertainty in the determination of κ

The uncertainty in $\kappa^{-1}$ is obtained by propagation of uncertainty analysis of Equation 2:

$$\Delta\kappa = \sqrt{\left(\frac{\partial\kappa}{\partial C_{indicator}}\right)^2 \Delta C_{base}{}^2 + \left(\frac{\partial\kappa}{\partial\delta_{obs}}\right)^2 \Delta\delta_{obs}{}^2 + \left(\frac{\partial\kappa}{\partial\delta_H}\right)^2 \Delta\delta_H{}^2 + \left(\frac{\partial\kappa}{\partial\delta_L}\right)^2 \Delta\delta_L{}^2} \quad \text{(S10)}$$

Where Δ indicates the uncertainty in the variable. Differentiating equation 2 with respect to each variable:

$$\frac{\partial\kappa}{\partial C_{indicator}} = \frac{\delta_{obs} - \delta_L}{\delta_H - \delta_L} \quad \text{(S11)}$$

$$\frac{\partial\kappa}{\partial\delta_{obs}} = \frac{C_{indicator} \times \delta_L}{\delta_H - \delta_L} \quad \text{(S12)}$$

$$\frac{\partial\kappa}{\partial\delta_H} = \frac{C_{indicator} \times (\delta_L - \delta_{obs})}{(\delta_H - \delta_L)^2} \quad \text{(S13)}$$

$$\frac{\partial\kappa}{\partial\delta_L} = -\frac{C_{indicator} \times (\delta_H - \delta_{obs})}{(\delta_L - \delta_H)^2} \quad \text{(S14)}$$

$$\Delta\kappa^{-1} = \sqrt{\left(\frac{\partial\kappa^{-1}}{\partial C_{indicator}}\right)^2 \Delta C_{base}{}^2 + \left(\frac{\partial\kappa^{-1}}{\partial\delta_{obs}}\right)^2 \Delta\delta_{obs}{}^2 + \left(\frac{\partial\kappa^{-1}}{\partial\delta_H}\right)^2 \Delta\delta_H{}^2 + \left(\frac{\partial\kappa^{-1}}{\partial\delta_L}\right)^2 \Delta\delta_L} \quad \text{(S15)}$$

Differentiating the inverse of equation 2 with respect to each variable:

$$\frac{\partial\kappa^{-1}}{\partial\delta_H} = \frac{1}{(\delta_{obs} - \delta_L) \times C_{indicator}} \quad \text{(S16)}$$

$$\frac{\partial\kappa^{-1}}{\partial\delta_L} = \frac{\delta_H - \delta_{obs}}{C_{indicator} \times (\delta_L - \delta_{obs})^2} \quad \text{(S17)}$$

$$\frac{\partial \kappa^{-1}}{\partial \delta_{obs}} = \frac{\delta_L - \delta_H}{C_{indicator} \times (\delta_{obs} - \delta_L)^2} \quad \text{(S18)}$$

$$\frac{\partial \kappa^{-1}}{\partial C_{base}} = \frac{\delta_L - \delta_H}{(\delta_{obs} - \delta_L) \times C_{indicator}{}^2} \quad \text{(S19)}$$

$\Delta\delta_{obs}$, $\Delta\delta_{L,}$ $\Delta\delta_H$ are taken as 0.0007 ppm in this work. $\Delta C_{indicator}$ is taken as 5% of expected concentration. As the pH in our experiments is within one unit of the p$K_a$ of the indicator, we can ignore uncertainty in pH as this is expected to be negligible in comparison to $\Delta\kappa^{-1}$ (Section S3).

## S5. Optimum time determination

In our previous work we demonstrated how to calculate the optimum time, $t_{op}$, as follows: The bases used in this work are highly water-soluble and dissolve within minutes of placing the analyte solution on top. Mathematically, the base behaves as though it were diffusing from a plane source at the bottom of the NMR tube. The concentration, $C_Z$, of diffusing base at a height Z from the bottom of the NMR tube after a time, t, is therefore given by equation S20:

$$C_z = N_{(t)}\, e^{(\frac{-(Z-h)^2}{4Dt})} \quad \text{(S20)}$$

Where D is the diffusion coefficient of the acid and N(t) is a time dependent parameter. h is the thickness of the solid base when placed at the base of the NMR tube (2 mm).

Integrating equation S20 over the length of the sample gives equation S21:

$$\mathrm{m} = \pi \mathrm{r}^2 \mathrm{a} \mathrm{C}_0 \mathrm{M}_\mathrm{r} \sqrt{\pi \mathrm{D} \mathrm{t}_{\mathrm{op}}}\, \mathrm{e}^{(\frac{(\mathrm{Z_a}-\mathrm{h})^2}{4\mathrm{D}\mathrm{t}_{\mathrm{op}}})} \quad \text{(S21)}$$

Where r is the radius of the NMR tube and Mr is the molecular weight of the base, a is the number of equivalents of diffusing basic indicator to acid at height $Z_a$ (18 mm). $C_0$ is the concentration of acidic species. Values for $t_{op}$ and D are shown in table S2. D values were estimated *via* Stokes–Einstein Gierer-Wirtz Estimation (SEGWE) method.[51] To determine $t_{op}$, for an experiment, $t_{op}$ was adjusted iteratively until m matched the mass of indicator weighed into the NMR tube (4-5 mg).

**Table S2.** D and $t_{op}$ values of indicators used

| Analyte | Indicator | D of indicator/$10^{-9}$ $m^2s^{-1}$ | $t_{op}$/hours |
|---|---|---|---|
| Glycine hydrochloride | 1,2,4-triazole | 0.88 | 4.73 |
| $H_3PO_4$ | 1,2,4-triazole | 0.88 | 4.73 |
| 4-cyanophenol,boric acid , $NH_4Cl$, $NaH_2PO_4$ | 2-methylimidazole | 0.81 | 5.53, 5.39, 5.33, 5.30[a] |
| $NH_3OHCl$ | 2,6-lutidine | 0.71 | 6.62 |
| Benzoic acid | Acetate | 0.81 | 5.32 |
| Glycolic acid | Formate | 0.90 | 4.62 |

[a] Analytes that were studied with 2-methylimidazole were grouped together with their respective $t_{op}$

## S6. Integral correction factors

To calculate the concentration of the base indicator across the NMR tube, a known concentration of either dioxane or DMSO are inserted into the analyte solution and the integral of one of the peaks of the base indicator is referenced to the peaks of DMSO or dioxane. A correction factor needs to consider the different number of protons for each respective peak along with imperfections due to incomplete $T_1$ relaxation. The correction factor is measured by preparing an $H_2O$ solution with a known concentration of 10 mM of the base indicator and 0.01% concentration of DMSO or dioxane with DSS as a chemical shift reference. A $^1$H 2D CSI experiment is then run on the sample immediately and the integrals of DMSO or Dioxane and the base indicator are measured. Correlating integral information and concentration is obtained using equation S22:

$$C_x = \frac{I_x}{I_{cal}} \times \frac{N_{cal}}{N_x} \times C_{cal} \times \rho \tag{S22}$$

where I, N, ρ, and C are the integral area, number of nuclei, imperfections due to incomplete $T_1$ relaxation, and concentration of the compound of interest (x) and the calibrant (cal), respectively. All compounds used in the present work had ≥ 98 % purity.

$\frac{N_{cal}}{N_x} \times C_{cal} \times \rho$ are defined as correction factor λ and equation S22 can be rearranged to obtain λ:

$$C_x \times \frac{I_{cal}}{I_x} = \lambda \tag{S23}$$

Table S3 displays correction factors for the indicators used in our experiments (Some indicators had only a correction factor of one of the reference compounds). Correction factor used was an average of the factors calculated for each experimental slice. Table S4 shows which integral references were used for each analyte choice was based on which integral reference gave least amount of spectral overlap.

**Table S3.** Indicator's correction factor for DMSO and Dioxane when applicable

| **Indicator** | **$\lambda$ (Dioxane)** | **$\lambda$ (DMSO)** |
|---|---|---|
| 2-methylimidazole (aromatic) | 4.69 | 5.43 |
| 2-methylimidazole (methyl) | 2.51 | 2.86 |
| 2,6-lutidine (methyl) | 1.37 | N/A due to signal overlap |
| Acetate | 2.14 | 2.90 |
| Formate | 9.45 | 12.73 |
| 1,2,4-triazole | 5.74 | 5.59 |

**Table S4.** Table showing which integral reference was used for each analyte

| **Dioxane** | **DMSO** |
|---|---|
| $NaH_2PO_4$ | $H_3PO_4$ |
| 4-CN | glycine hydrochloride |
| Boric acid | Benzoic acid |
| $NH_4Cl$ | Glycolic acid |
| $NH_3OHCl$ | Lectin |
| PAA | |

## S7. Determining limiting chemical shifts of indicators

The limiting chemical shifts of formate, acetate, 1,2,4-triazole and 2-methylimidazole in table 1 were determined by running an NMR experiment in which 4-5 mg of solid base indicator was weighed into the tube. Four, 2 mm diameter glass beads (Assistent, Germany) were then placed on top of the base. An aliquot of an $H_2O$ solution containing a 10 mM concentration of HCl, 0.2 mM DSS and 0.01% DMSO was drawn up in a 9″ Pasteur pipet and gently layered on top of the glass beads to a height of 40-50 mm from the base of the NMR tube. A $^1H$ 2D CSI experiment was then run on the sample after 4-6 hours.

DSS and DMSO are used as chemical shift reference and integral reference respectively. Equation 3 approximates $\kappa$ to $C_{analyte}$ as p$K_a$ decreases (This is true only when $C_{indicator}$ >> $C_{analyte}$ as $\kappa$ cannot be greater than $C_{indicator}$). Equation 3 can be reorganised to produce equation S24 where a linear plot $\kappa/C_{indicator}$ versus $\delta_{obs}$ is produced (Figure S2) with the slope as $(\delta_H-\delta_L)^{-1}$ and the intercept as $(-\delta_L/(\delta_H-\delta_L))$. Hence, through simple algebraic manipulation $\delta_H$ and $\delta_L$ can be calculated. Concentration of base needs to be higher than the acid as $\kappa$ cannot be bigger than the concentration of base.

$$\kappa = \frac{C_{analyte}}{1+10^{pKa-pH}} \approx C_{analyte} \text{ when HCl is the acidic analyte } (pK_a << 0)$$

$$\frac{\kappa}{C_{indicator}} = \frac{\delta_{obs}}{\delta_H - \delta_L} - \frac{\delta_L}{\delta_H - \delta_L} \quad \text{(S24)}$$

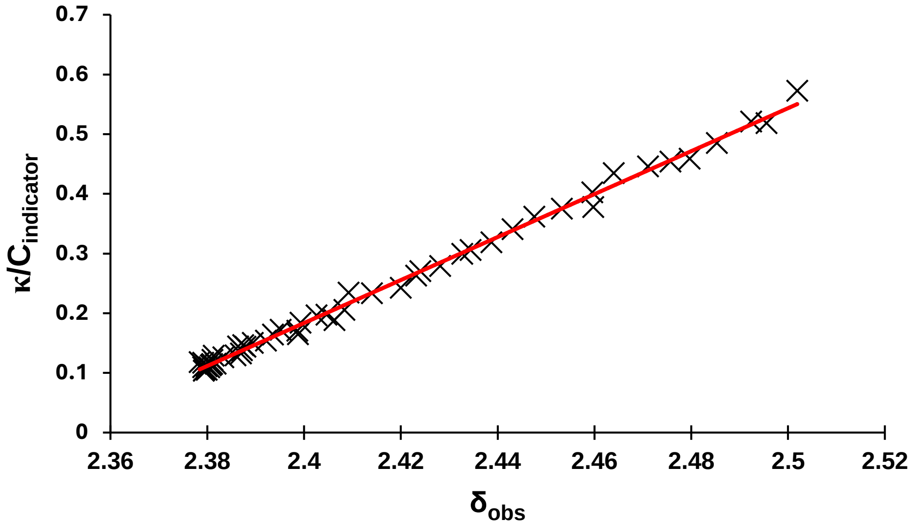


**Figure S2.** Plot of $\kappa/C_{indicator}$ versus $\delta_{obs}$ for 2-methylimidazole with 10 mM HCl in $H_2O$ solution.

## S8. 2D $^1$H NMR chemical shift spectra array of sodium dihydrogen phosphate

2D spectra of sodium dihydrogen phosphate are shown on figure S3. The change in chemical shift of 2-methylimidazole is seen as a sigmoidal curve, additionally the spectra highlights the absence of chemical shift of sodium dihydrogen phosphate. pH and κ is measured by tracking how the chemical shift of 2-MI changes across the sample (see Figure S4).

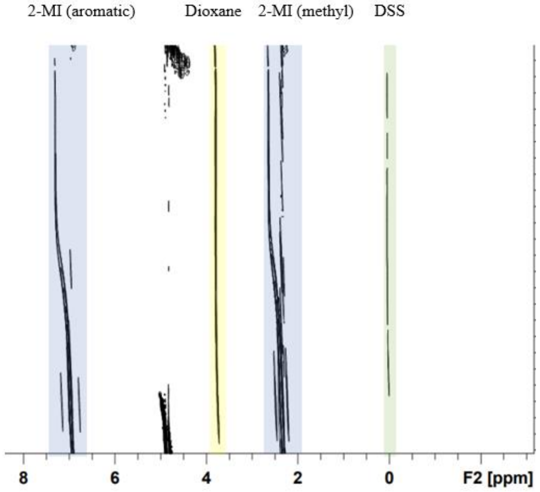


**Figure S3.** 2D CSI $^1$H plot of 10 mM sodium dihydrogen phosphate $H_2O$ solution with 0.01% Dioxane, 0.2 mM DSS and 4.7 mg of 2-methyl imidazole (2-MI) as diffusing base.

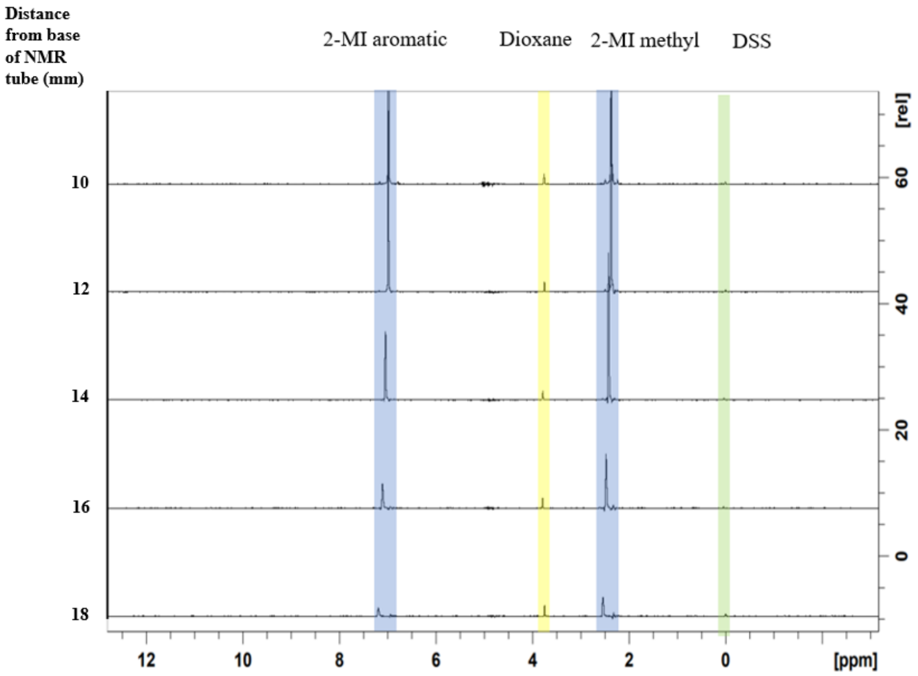


**Figure S4.** Stacked $^{1}$H NMR spectra of sodium dihydrogen phosphate spectra across the NMR tube

## S9. $^{1}$H NMR spectra of homogenous polyacrylic acid with sodium acetate, and concentration gradients of $NH_3$ and NaCl

In homogenoues mixtures of PAA (10 mM COOH groups) and sodium acetate, the $^{1}$H resonances of PAA exhibit no difference in intensity between 5 and 200 mM sodium acetate (Figure S5), although the resonances are shifted slightly upfield at the higher concentration due to deprotonation. These samples contained 0.2 mM DSS and 0.01% dioxane and were otherwise identical to the sample of Figure 2. The loss of the PAA resonances observed on Figure 2 is thus attributable to an effect of the concentration gradient of sodium acetate. To further investigate the origin of this effect, a sample was prepared by layering 60 μL of 0.5 M $NH_3$ on top of 500 μL of PAA solution to create a concentration gradient of $NH_3$, with a higher concentration of base towards the top of the sample, in contrast to the sodium acetate. Significant loss of intensity of PAA is observed towards the top of the sample (Figure S6). A sample prepared analgously to Figure 2, but with NaCl in place of sodium acetate, exhibits no change in the intensity of the PAA resonances along its length, suggesting diffusiophoresies along a salt gradient is not the source of signal loss of the PAA (Figure S7).

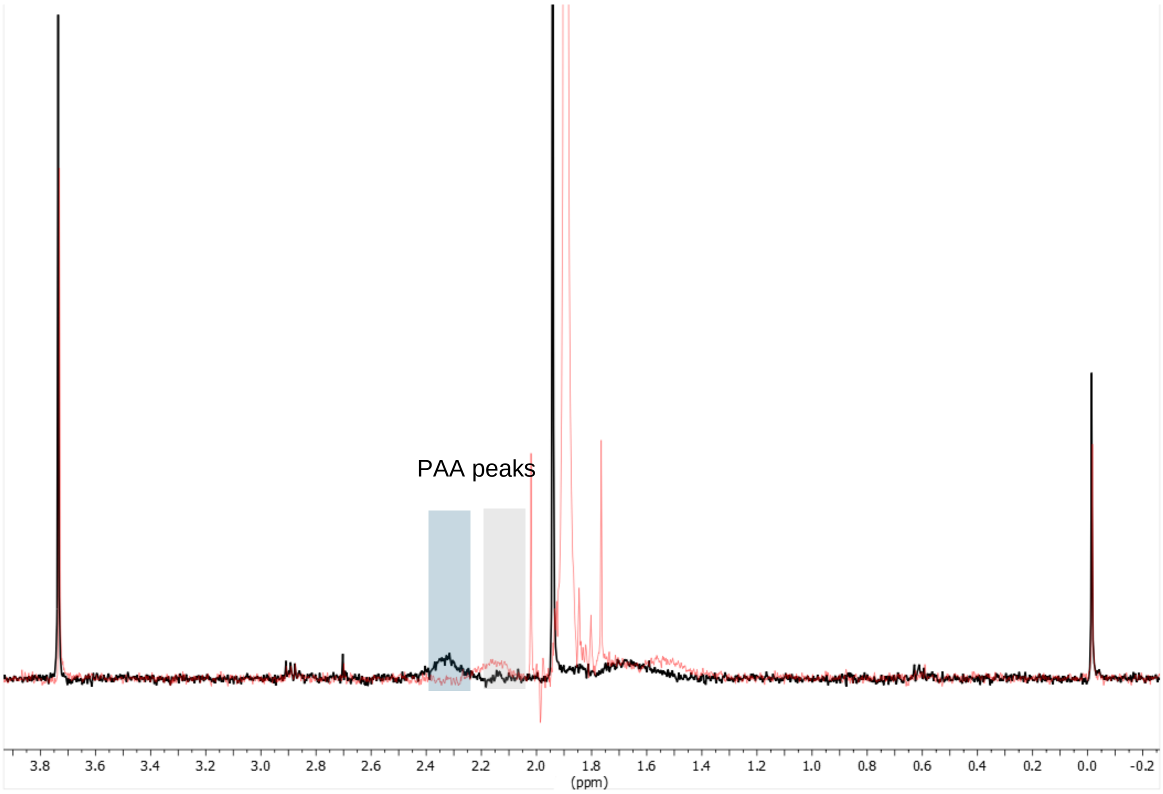


**Figure S5.** Stacked $^{1}$H NMR spectra of homogenous polyacrylic acid solutions 910 mM COOH groups) with 200 mM sodium acetate (red spectrum), 5 mM sodium acetate (black spectrum).

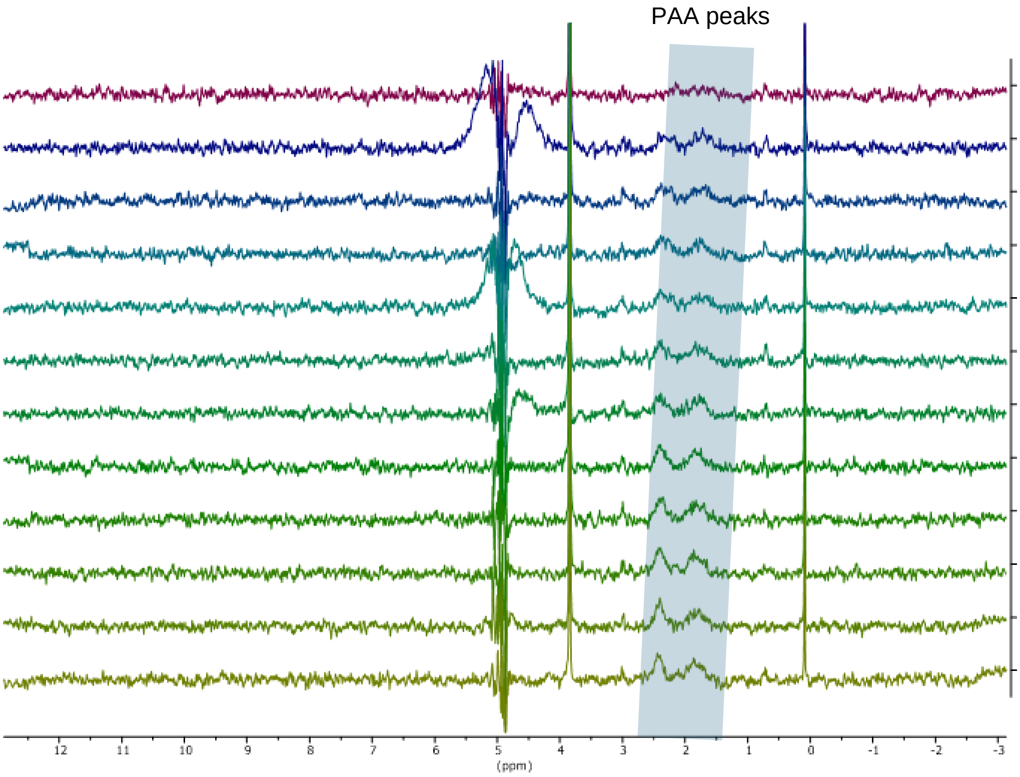


**Figure S6.** Stacked $^{1}$H NMR spectra of 10 mM polyacrylic acid solution with concentration gradient of ammonia (the top slices correspond to the top of the NMR active region of the sample tube, closer to the source of the ammonia).

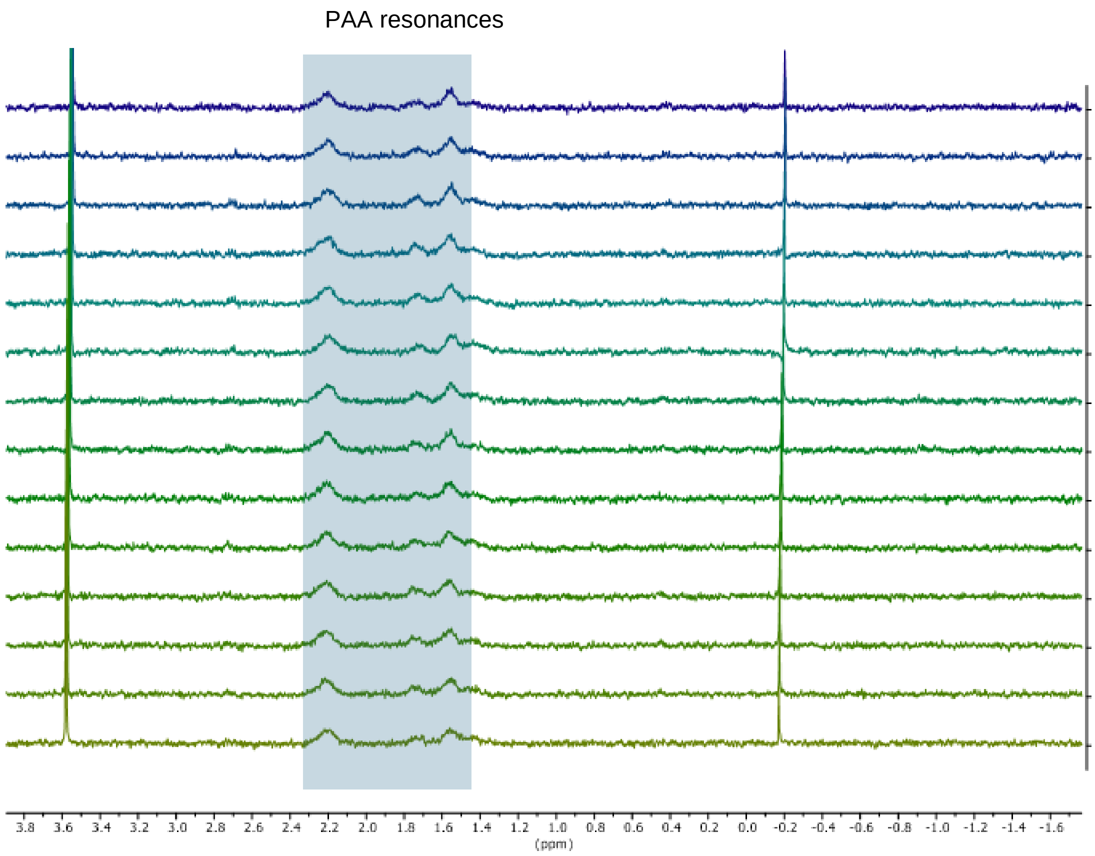


**Figure S7.** Stacked $^1$H NMR spectra of 10 mM polyacrylic acid solution with NaCl gradient (the top slices correspond to the top of the NMR active region of the sample tube, with a lower concentration of NaCl at the top).

## S10. Alternative method for determining p$K_a$ and concentration of analyte

p$K_a$ and $C_{analyte}$ can be determined directly from equation 2 and 3 by obtaining κ using equation 3 and then fitting the measured κ using equation 3 where p$K_a$ and $C_{analyte}$ are used as free parameters with pH measured using indicators and equation 1 (displayed are all the equations relevant). The fitting is done using excel through nonlinear generalised reduced gradient function (GRG), which looks at the gradient of the objective function as the input values are changed and determines whether the partial derivatives are equal to the values desired. In the context of p$K_a$ measurement, the objective function is set to equal 1 and the objective function is an $R^2$ comparison between the pH obtained from the modified Henderson-Hasselbalch equation from an indicator compound and the analyte in question (Example is shown in figure S8 for the method and its respective result).

$$pH = \mathrm{p}K_{\mathrm{a},0} + \log\frac{\delta_{\mathrm{obs}} - \delta_{\mathrm{H}}}{\delta_{\mathrm{L}} - \delta_{\mathrm{obs}}} + \Delta z^2\left(\frac{0.51\sqrt{\mathrm{I}}}{1+\sqrt{\mathrm{I}}} - 0.1\mathrm{I}\right) \quad (2)$$

$$\kappa = C_{indicator}\,\frac{\delta_{obs} - \delta_L}{\delta_H - \delta_L} \quad (3)$$

$$\kappa = \frac{C_{analyte}}{1 + 10^{pKa-pH}} \qquad (3)$$

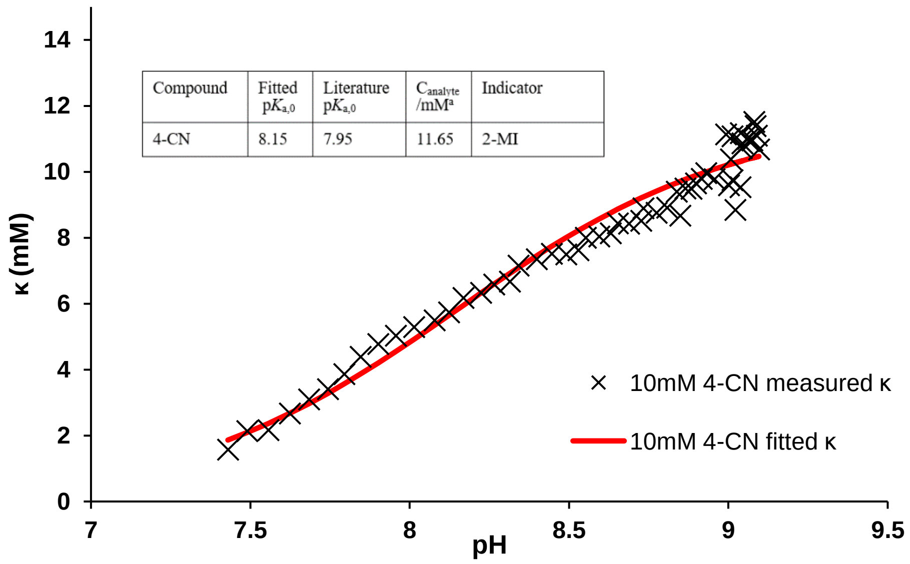


**Figure S8.** Κ versus pH graph of 10mM 4-CN with 2-MI as indicator, both measured κ and fitted κ are displayed ($R^2$ of 0.96 is obtained)

## S11. Impact of $H_2O$ on protonation of indicators

Taking 2-methylimidazole as the indicator with highest p$K_a$ and hence most susceptible to protonation from $H_2O$. Calculation of fraction of indicators protonated $f_H$ from $H_2O$ we use the NMR modified Hendersson-Hasselbalch equation S8 and 1:

$$\mathrm{pH} = \mathrm{pK_{a,0}} + \log_{10}\left(\frac{\delta_{obs} - \delta_H}{\delta_L - \delta_{obs}}\right) + \Delta z^2\left(\frac{0.51\sqrt{I}}{1+\sqrt{I}} - 0.1I\right) \qquad (1)$$

$$\mathrm{pH} = \mathrm{pK_{a,0}} + \log_{10}\left(\frac{f_{H\,indicator}}{f_{L\,indicator}}\right) = \mathrm{pK_{a,0}} + \log_{10}\left(\frac{f_{H\,indicator}}{1 - f_{H\,indicator}}\right) \qquad (S8)$$

Nine Separate 1D proton NMR experiments were run each containing 2-methylimidazole in $H_2O$ with respective concentrations of 1, 2, 4, 5, 10, 20, 50, 100 and 200 mM. pH of solution in all experiments was measured using equation 1. $f_H$ of 2-methylimidazole for each experiment was calculated using S25:

$$f_{H\,indicator} = \frac{1}{1 + 10^{\mathrm{pK_{a,0}} - \mathrm{pH}}} \qquad (S25)$$

Figure S9 demonstrates how the fraction protonated of 2-methylimdazole from $H_2O$ is 3.22 % when concentration of 2-methylimidazole is 1 mM and $f_H$ decays in a sigmoidal fashion as concentration of 2-methylimidazole increases. This implies that impact of protonation from $H_2O$ to 2-methylimidazole is negligible and hence impact of protonation from water to indicators that are of lower p$K_a$ than 2-methylimidazole is negligible as well.

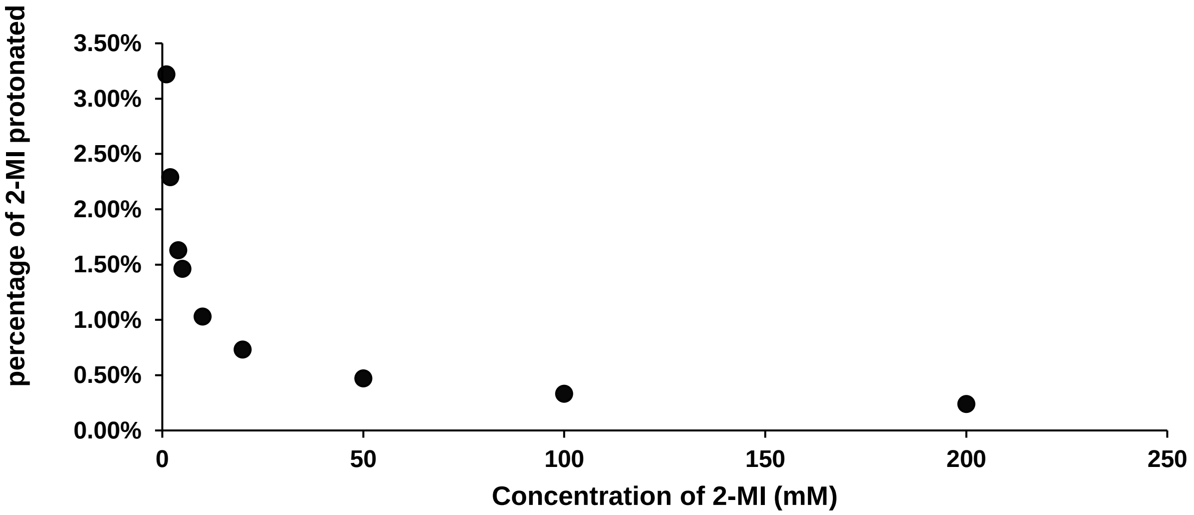


**Figure S9.** Figure showing how $f_H$ of 2-MI changes in water as a function of its concentration

## S12. Determining the protons transferred to $H_2O$ for low p$K_a$ determination

Working with analytes with relatively low p$K_a$ (such as phosphoric acid) requires the use of a relatively weak base (such as 1,2,4-triazole) and due to the weak basicity of the base the quantiy of protons transferred from the acidic analyte to water becomes significant and can not be ignored. To determine the protons transferred from the acidic analyte neccesitates measuring the quantiy of protons transferred to the basic indicator and to $H_2O$. Determining the quantity of protons transferred to $H_2O$ is done simply by measuring the concentration of hydronium ions (which is just $10^{-pH}$ ).

$$\mathbf{C}_{\mathbf{analyte}}\mathbf{f}_{\mathbf{L\ analyte}} = \mathbf{C}_{\mathbf{indicator}}\ \mathbf{f}_{\mathbf{H\ indicator}} + \mathbf{10}^{-\mathbf{pH}}$$

## S13. Plot of 1/κ versus pH for all analytes

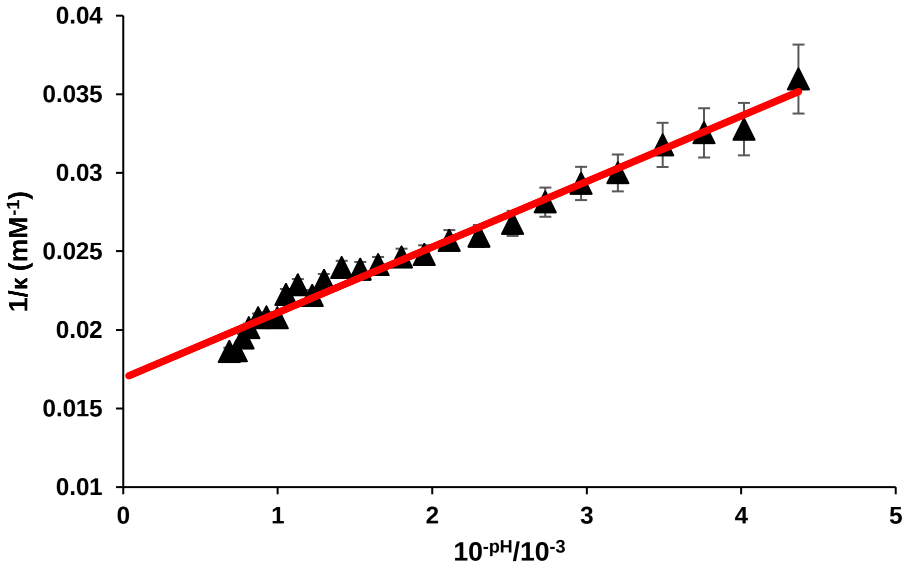


**Figure S10.** Plot of 1/κ versus $10^{-pH}$ for $H_3PO_4$ with 1,2,4-triazole as indicator

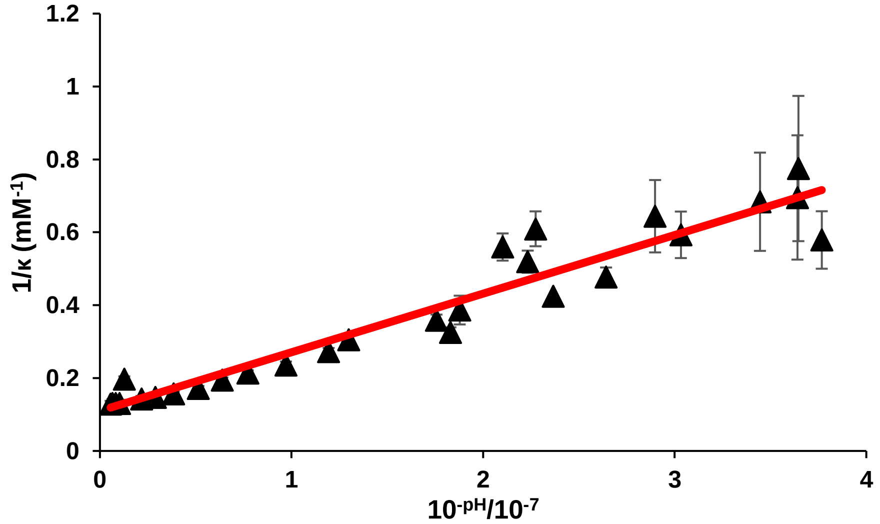


**Figure S11.** Plot of 1/κ versus $10^{-pH}$ for $NaH_2PO_4$ with 2-methylimidazole as indicator

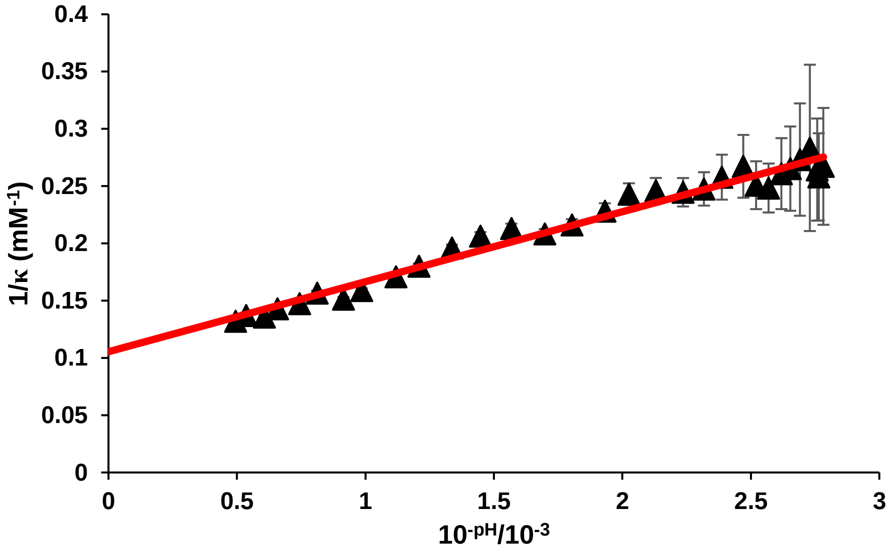


**Figure S12.** Plot of 1/κ versus $10^{-pH}$ for Glycine hydrochloride with 1,2,4-triazole as indicator

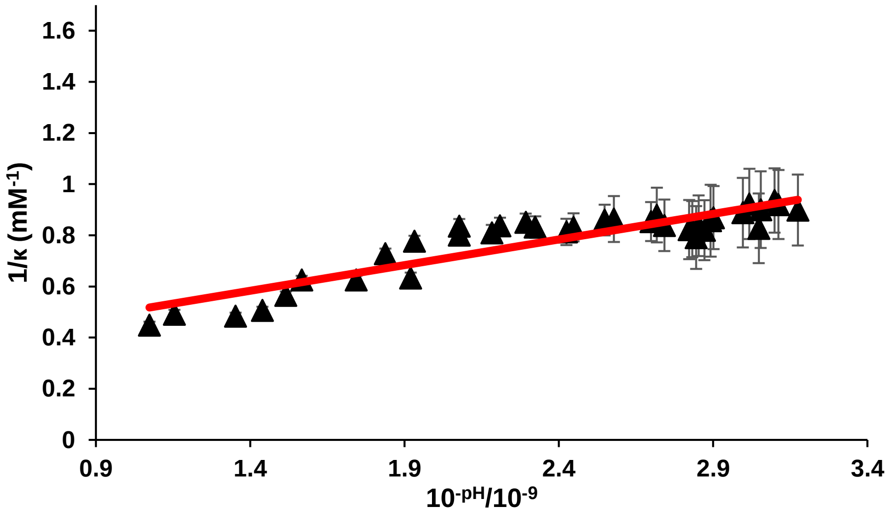


**Figure S13.** Plot of 1/κ versus $10^{-pH}$ for $NH_4Cl$ with 2-methylimidazole as indicator

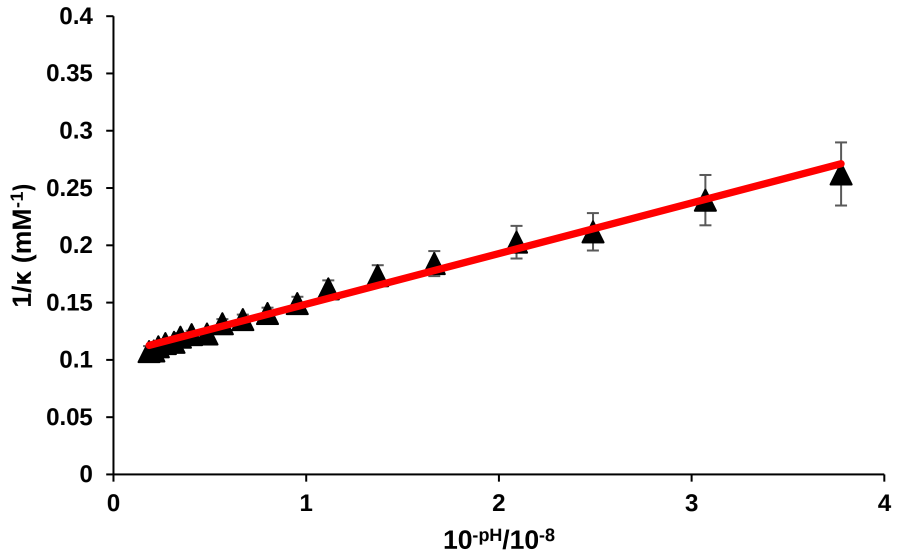


**Figure S14.** Plot of 1/κ versus $10^{-pH}$ for 4-cyanophenol  with 2-methylimidazole as indicator

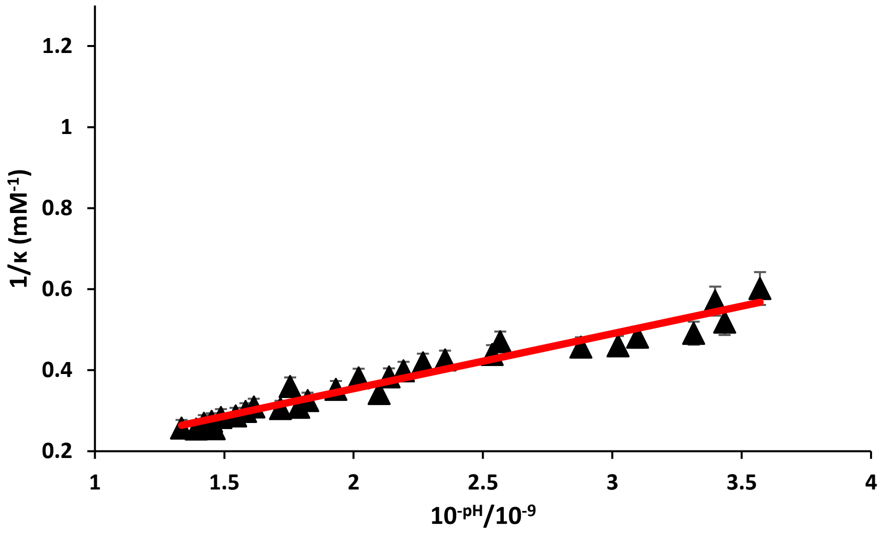


**Figure S15.** Plot of 1/κ versus $10^{-pH}$ for Boric acid  with 2-methylimidazole as indicator

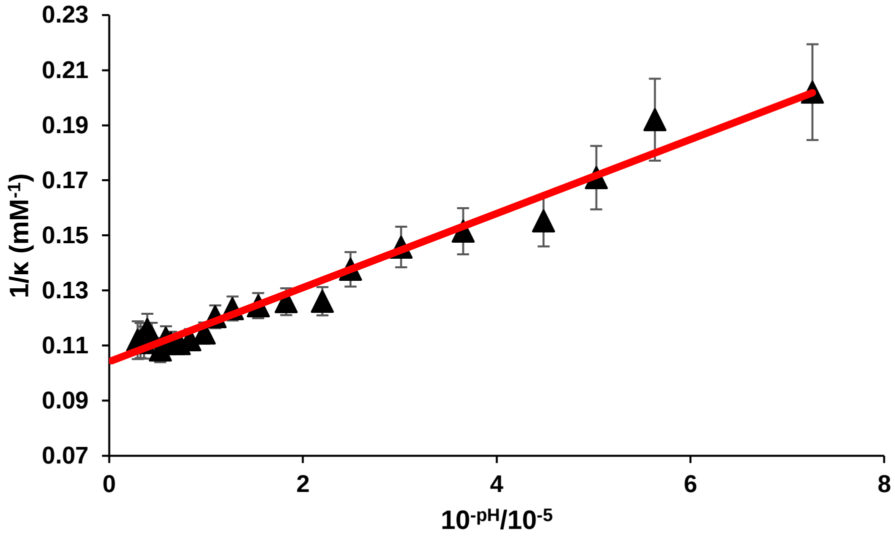


**Figure S16.** Plot of 1/κ versus $10^{-pH}$ for benzoic acid  with sodium acetate as indicator

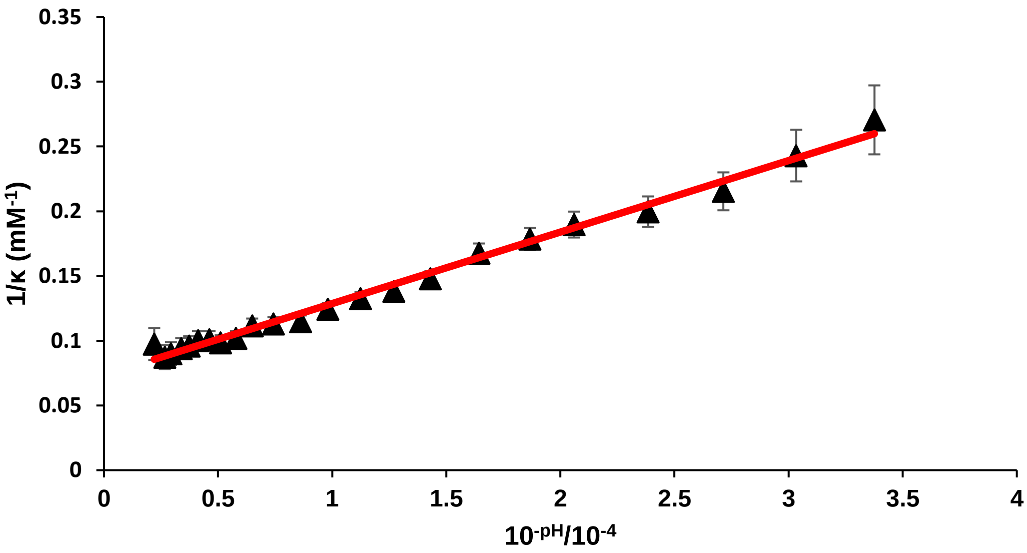


**Figure S17.** Plot of 1/κ versus $10^{-pH}$ for glycolic acid  with sodium formate as indicator

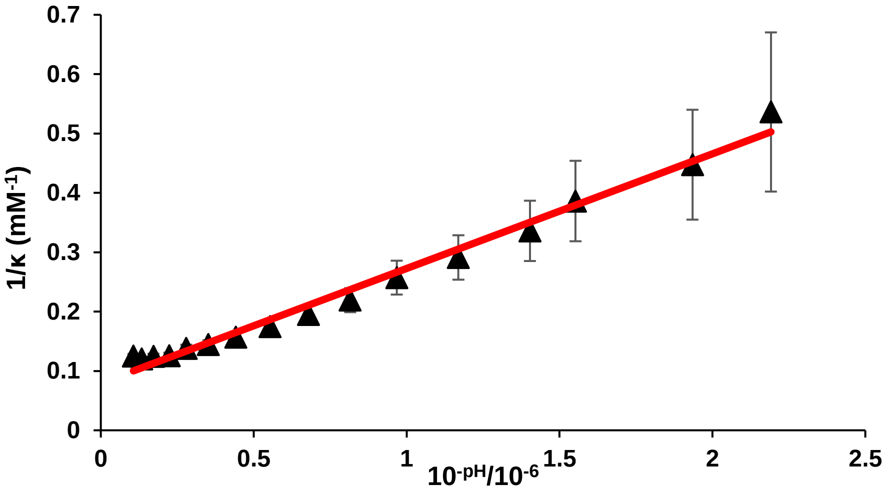


**Figure S18.** Plot of 1/κ versus $10^{-pH}$ for $NH_3OHCl$ with 2,6-lutidine as indicator

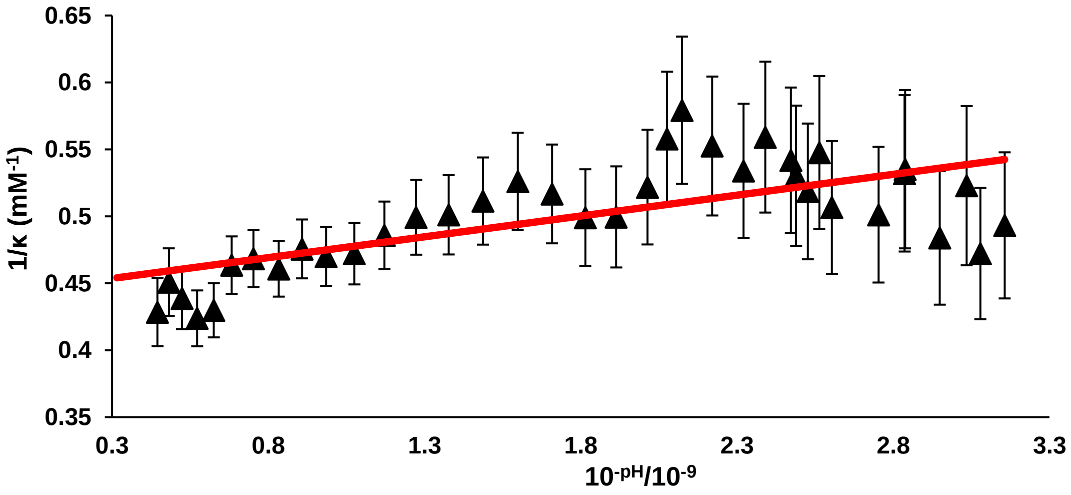


**Figure S19.** Plot of 1/κ versus $10^{-pH}$ for WGA with 2-methylimidazole as indicator

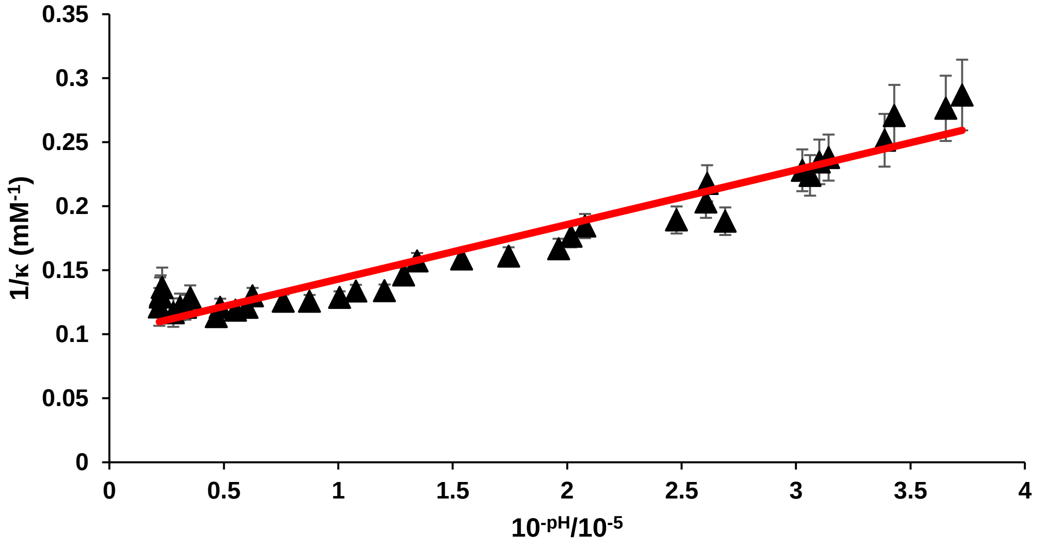


**Figure S20.** Plot of 1/κ versus $10^{-pH}$ for polyacrylic acid with sodium acetate as indicator

49. Szakács, Z.; Hägele, G.; Tyka, R., 1H/31P NMR pH indicator series to eliminate the glass electrode in NMR spectroscopic pKa determinations. *Anal. Chim. Acta.* **2004,** *522* (2), 247-258.
50. Robinson, R. A.; Stokes, R. H., *Electrolyte solutions*. Courier Corporation: 2002.
51. Tang, B.; Chong, K.; Massefski, W.; Evans, R., Quantitative Interpretation of Protein Diffusion Coefficients in Mixed Protiated-Deuteriated Aqueous Solvents. *J Phys Chem B* **2022,** *126* (31), 5887-5895.

# S14. 2D pulse sequence for CSI (Bruker)

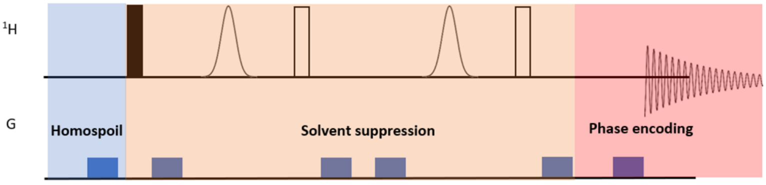


**Figure S21.** Schematic to show chemical shift imaging sequence utilised in this work. It is broken down into three sections: (blue) homospoil gradient to destroy transverse magnetisation remaining from previous scan, (orange) solvent suppression sequence through excitation sculpting, (red) phase encoding sequence

```
;Modified from: "Probing spatial distribution of alignment by deuterium NMR imaging"
;Chemistry - A European Journal, Volume 19, Issue 22, 27 May 2013, Pages 7013-7019
;2D sequence for z-imaging preserving chemical shift
;using a phase encoding gradient. Original sequence written by Christian Merle, Martin Koos
;Modified to be on 1H with Excitation sculpting for water suppression
;Matthew Wallace, 3/2022 (University of East Anglia, matthew.wallace@uea.ac.uk)
;This pulse program is not fully tested and comes without warranty.
;!!This version is for running unlocked with sweep etc. disabled
;!!For running locked, delete the (UN)BLKGRAMP and insert the (UN)BLKGRAD
;Set 1 SW to Z-range in mm (see cnst0) to get 1 Hz/mm scale in indirect dimension
;Make cnst0 bigger than actual sample size to avoid folding artefacts.
;Keep gpz6 at 100% and adjust cnst3 to get p30 to an acceptable length according to instrument (ca. 150-300 us)
; 1H-Version
;$CLASS=HighRes
;$DIM=2D
;$TYPE=
;$SUBTYPE=
;$COMMENT=
prosol relations=<triple>
#include <Avance.incl>
#include <Grad.incl>
#include <Delay.incl>
"cnst2= 0.8914027" ; integralfactor of gradient shape SMSQ10.32
"cnst4= 267.52220" ; * 10^6 /Ts = gamma1H
"p30=(td1/cnst0)*(1/(cnst1*cnst2*cnst3))*(1/cnst4)*(2*3.14159265/1000)*0.5 s"
"l1=td1-1"
lgrad r1d = l1
"acqt0=0"
"DELTA1=p30+d16"
"TAU=de+p1*2/3.1416+50u"
"p2=p1*2"
"d12=20u"
"d4=d1-100m"
baseopt_echo
1 ze
2 30m ;BLKGRAD so locked during d1
d1
;spoil gradient from previous acquisition
3 50u UNBLKGRAMP ;UNBLKGRAD
p19:gp3
d16
;start of zgesgp
d12 pl1:f1
 p1 ph1
 50u
 p16:gp1
 d16 pl0:f1
 (p12:sp1 ph2:r):f1
 4u
 d12 pl1:f1
 p2 ph3
 4u
 p16:gp1
 d16
 TAU
DELTA1
 p16:gp2
 d16 pl0:f1
 (p12:sp1 ph4:r):f1
 4u
 d12 pl1:f1
 p2 ph5
 4u
 p16:gp2
 d16
p30:gp6*cnst3*r1d
S32
d16 BLKGRAMP ;remove this BLKGRAMP statement if running locked
go=2 ph31
30m
100m wr #0 if #0 zd igrad r1d
d4
lo to 3 times l1
goto 5
; run last increment:
4 30m ;BLKGRAD
d1
;spoil gradient from previous
5 50u UNBLKGRAMP ;UNBLKGRAD
p19:gp3
```

```
d16
 ;start of zgesgp
d12 pl1:f1
 p1 ph1
 50u
 p16:gp1
 d16 pl0:f1
 (p12:sp1 ph2:r):f1
 4u
 d12 pl1:f1
 p2 ph3
 4u
 p16:gp1
 d16
 TAU
DELTA1
 p16:gp2
 d16 pl0:f1
 (p12:sp1 ph4:r):f1
 4u
 d12 pl1:f1
 p2 ph5
 4u
 p16:gp2
 d16
p30:gp6*cnst3*r1d
d16 BLKGRAMP ;remove this BLKGRAMP statement if running locked
go=4 ph31
30m ;BLKGRAD
100m wr #0 if #0 zd
d3
exit
;Phase cycling as for zgesgp
ph1=0
ph2=0 1
ph3=2 3
ph4=0 0 1 1
ph5=2 2 3 3
ph31=0 2 2 0
;pl0 : 0W
;cnst0 : z-Range in cm
;cnst1 : GCC (G/mm) from Gradpar
;cnst3 : set max to get acceptable length of GP of ca 150
-300us [0.95 max]
;pl1 : f1 channel- power level for pulse (default)
;sp1 : f1 channel- shaped pulse 180 degree
;p1 : f1 channel- 90 degree high power pulse
;p12: f1 channel- 180 degree shaped pulse (Gaus1.1000) [4 msec]
;p16: homospoil/gradient pulse for water suppression (1000 us)
;p19 : Spoil gradient pulse (1000 us)
;gpz6: 100% phase encoding gradient
;d1 : Relaxation delay
;d3: waiting time before next experiment in a time delay sequence [s] set to 0.1 if no delay
;d12: delay for power switching [20 usec]
;d16: standard eddy delay (200u)
;ns: 4 * n, total number of scans: NS * TD0
;ds: 2*m [16]
;td1: number of experiments
;FnMODE: QF
;use gradient ratio: gp 1 : gp 2
; 31 : 11
;for z-only gradients:
;gpz1: 31%
;gpz2: 11%
;gpz3: 50%
;use gradient files:
;gpnam1: SMSQ10.100
;gpnam2: SMSQ10.100
;gpnam3: SMSQ10.100
;gpnam6: SMSQ10.32
;$Id: zgesgpimg,v 1.9 2012/01/31 17:49:32 ber Exp $
```

# S15. AU program to process raw CSI datasets

```
/*To produce phase corrected chemical shift image from gradient encoded data*/
/*Set 1st order phase correction for f1 dimension to 180*Td1 (11520 when 64 points in image), 0th order to 0*/
/*PH_mod should be set to PK in both dimensions*/
/*XFB to produce image*/
/*This is done automatically by script 13.2 above*/
/*With the 2D dataset selected, Run this Au*/
/*AU extracts each row in turn to a procno and automatically phase and baseline corrects/*
/*Reversal of F1 axis may be necessary, depending on NMR probe*/
/*This AU is not fully tested and comes without warranty.*/
/*The script works on Bruker Topspin 3.6.2 but has not been tested on other versions*/
/*Use kill command if all goes wrong*/
/*Matthew Wallace, 3/2022*/
/*University of East Anglia, matthew.wallace@uea.ac.uk*/
char disk1[32], user1[32], location[128], phtyp[8];
float abf1=8;
float abf2=6;
int phpno=1;
int w=1;
int np=64;
int pno=5;
GETCURDATA
int steno=expno;
strcpy(location,disk);
strcpy(phtyp,"k");
```

```
GETSTRING("Enter location of dataset",location)
phpno=procno;
GETINT("Enter experiment number to process",steno)
GETINT("Enter procno containing XFB processed 2D data :",phpno)
pno=phpno+5;
GETINT("Enter procno to write rows to phase and baseline correct (empty):",pno)
REXPNO(steno)
RPROCNO(phpno)
SETCURDATA
FETCHPAR1("SI",&np)
GETINT("Enter number of points in image (autodetects) :",np)
GETSTRING("APKS (s) or APK (k) or apkf (f) auto phase correction?",phtyp)
if(strcmp(phtyp,"f")==0)
{
GETFLOAT("Enter right limit for apkf and absf:",abf2)
GETFLOAT("Enter left limit for apkf and absf:",abf1)
}
w=1;
TIMES(np)
{
RPROCNO(phpno)
SETCURDATA
RSR(w,pno)
RPROCNO(pno)
SETCURDATA
        if(strcmp(phtyp,"s")==0)
        {
APKS
ABS
        }
        if(strcmp(phtyp,"k")==0)
        {
APK
ABS
        }
        if(strcmp(phtyp,"f")==0)
        {
                STOREPAR("absf1",abf1)
                STOREPAR("absf2",abf2)
APKF
ABSF
        }
        WSR(w,phpno,steno,name,user,location)
w++;
        }
        END
        QUITS
```

# S16. AU program to pick peak a chemical shift in CSI

```
/*Goes through a 2D image and writes file of peak position/ppm*/
/*The script works on Bruker Topspin 3.6.2 but has not been tested on other versions*/
/*Use kill command if all goes wrong*/
/*Matthew Wallace and Haider Hussain, 6/2024*/
/*University of East Anglia, matthew.wallace@uea.ac.uk*/
/*Will pick centre of a multiplet*/
FILE *find;
float min=0;
double f2pind=1.01;
double f1pind=1.6;
float ppsens=0.9;
double pc=0.1;
int steno=15;
int eno;
int ne=5;
int m=-1;
int rn=1;
double peakFreqHz, peakFreqPPM, peakIntensity, maxpsh, maxpsp, maxips, sf,sfo1,so1p,mintpp,minpsp,peakppmneg,cent,ppmdif,maxpspneg;
int i, numPeaks;
int np=64;
int row=1;
int v=1;
int phpno=1;
GETCURDATA
steno=expno;
phpno=procno;
FETCHPAR1("td",&np)
GETINT("Enter starting experiment number: ",steno)
GETINT("Enter number of gradient values",np)
GETINT("Enter procno of 2D dataset",phpno)
GETDOUBLE("Indictaor right peak picking/ppm",f2pind)
GETDOUBLE("Indicator left peak picking/ppm",f1pind)
GETFLOAT("Enter peak picking senistitivty factor",pc)
GETDOUBLE("Enter satelite sensitivity factor",ppsens)
/*Reads in peak picking parameters for ind*/
REXPNO(steno)
SETCURDATA
RPROCNO(phpno)
SETCURDATA
STOREPAR("mi",min)
STOREPAR("pc",pc)

if ((find = fopen(ACQUPATH("Indicator chemical shift.txt"),"wt")) == 0)
   STOPMSG("Cannot create difflist")
TIMES(np)
{
RSR(v,5)
RPROCNO(5)
SETCURDATA
STOREPAR("f2p",f2pind)
STOREPAR("f1p",f1pind)
PP
```

```
 numPeaks = readPeakList(PROCPATH(0));
maxips=0.0;
   maxpsh=0.0;
   for (i=0; i<numPeaks; i++)
   {
                 peakIntensity = getPeakIntensity(i);
                 peakFreqHz = getPeakFreqHz(i);
                 peakFreqPPM = getPeakFreqPPM(i);
                 if (peakIntensity > maxips)
                 {
                    maxips = peakIntensity;
                    maxpsh = peakFreqHz;
                    maxpsp = peakFreqPPM;
                 }
   }
   /*Pick most downfield side of multiplet*/
   mintpp=maxips*ppsens;
maxpsp=0.0;
   for (i=0; i<numPeaks; i++)
   {
                                 peakIntensity = getPeakIntensity(i);
                                 if(peakIntensity>mintpp)
                                 {
                 peakFreqPPM = getPeakFreqPPM(i);
                 peakFreqHz = getPeakFreqHz(i);
                 if (peakFreqHz >= maxpsh)
                 {
                    maxpsp = peakFreqPPM;
                    maxpsh = peakFreqHz;
                 }
   }
                 }
/*Flips negative to choose most upfield peak of multiplet*/
for (i=0; i<numPeaks; i++)
   {
                                 peakIntensity = getPeakIntensity(i);
                                 if(peakIntensity>mintpp)
                                 {
                 peakFreqPPM = getPeakFreqPPM(i);
peakppmneg=peakFreqPPM*m;
maxpspneg=maxpsp*m;
                 if (peakppmneg >= maxpspneg)
                 {
                    minpsp = peakFreqPPM;
                 }
   }
                 }
   freePeakList();
/*writes centre of multiplet into text document*/
ppmdif=maxpsp-minpsp;
cent=minpsp+ppmdif*0.5;
   fprintf(find,"%f \n",cent);
v++;
rn++;
RPROCNO(phpno)
SETCURDATA
}
END
fclose(find);
QUIT
```

# S17. AU program to integrate a peak in CSI

```
/*Bruker AU script for integrating a CSI dataset*/
/*CSI dataset should have been fully processed in phase-sensitive mode*/
/*Crude chemical shift referencing in F2 also helps*/
/*The script works on Bruker Topspin 3.6.2 but has not been tested on other versions*/
/*Create a 1D integral file using the wmisc command*/
/*This should contain only the reference compound and indicator*/
/*Copy and paste this name into the text box when asked, or paste into strcpy(text,"indref"); line 46 below, in place of indref */
/*indref is the name of the integral range file used in this script by default*/
/*The script extracts each row in turn into the empty procno requested (will overwrite existing contents!!!)*/
/*Working in this procno, the spectrum is referenced to the chemical shift reference supplied*/
/*Default values here apply to DMSO, DMSO signal is downfield of indicator (ordans=d)*/
/*Change right and left peak picking limits if any risk of a non-reference peak being included in the referencing procedure*/
/*Having referenced the spectrum, the requested integral file is read in and the integral values exported to .txt documents*/
/*Each .txt document is stored in the procno directory of the 2D CSI dataset*/
/*The script will also by default save rows 15, 32 and 50 into procnos of the same number*/
/*This is so the work of the script can be checked - spectra are correctly referenced and integrals correctly applied*/
/*This AU is not fully tested and comes without warranty.*/
/*Use kill command if all goes wrong*/
/*Matthew Wallace and Haider Hussain, 6/2024*/
/*University of East Anglia, matthew.wallace@uea.ac.uk*/
FILE    *fpnt,*fref,*fac;
char savans[8],dummystr[256],intdir[256],location[128],ordans[8];
float min=0;
double f2pdss=-0.5;
double f1pdss=0.5;
double ref=0;
float ppsens=0.9;
double pc=0.1;
int m=-1;
int rn=1;
int sav1=15;
int sav2=32;
int sav3=50;
int steno;
double peakFreqHz, peakFreqPPM, peakIntensity, maxpsh, maxpsp, maxips, sf,sfn,sfo1,intgr;
```

```
double intnum,ppmdn,ppmup,intgrso1p,mintpp,minpsp,peakppmneg,cent,ppmdif,maxpspneg;
int i, numPeaks;
int np=64;
int row=1;
int v=1;
int wrpno=5;
int linenum=1;
int phpno=1;
strcpy(location,disk);
strcpy(savans,"y");
strcpy(text,"indref");
strcpy(ordans,"d");
GETCURDATA
steno=expno;
phpno=procno;
GETINT("Enter experiment number of 2D dataset",steno)
GETINT("Enter procno of 2D dataset",phpno)
REXPNO(steno)
RPROCNO(phpno)
SETCURDATA
FETCHPAR1("td",&np)
GETINT("Enter number of spectra in CSI image",np)
GETINT("Enter procno to extract rows into for integration (blank)",wrpno)
GETDOUBLE("Chemical shift reference right peak picking/ppm",f2pdss)
GETDOUBLE("Chemical shift reference left peak picking/ppm",f1pdss)
GETDOUBLE("Enter reference shift/ppm",ref)
GETFLOAT("Enter peak picking sensitivity factor",pc)
GETDOUBLE("Enter satellite sensitivity factor",ppsens)
GETSTRING("Save example spectra to check working OK? y/n",savans)
if(strcmp(savans,"y")==0)
{
GETINT("Enter 1st spectrum to save (put in pno of same#)",sav1)
GETINT("Enter 2nd spectrum to save (put in pno of same#)",sav2)
GETINT("Enter 3rd spectrum to save (put in pno of same#)",sav3)
}
GETSTRING("Which intrng file must be used?", text)
GETSTRING("Is the reference integral upfield (u) or downfield (d) of indicator?",ordans)
REXPNO(steno)
SETCURDATA
RPROCNO(phpno)
SETCURDATA
STOREPAR("mi",min)
STOREPAR("pc",pc)
STOREPAR("CURPRIN","Integrals.txt")
/*Create text files to hold integral data*/
/*Global scaling*/
STOREPAR("pscal",0)
if ((fref = fopen(ACQUPATH("Reference integral.txt"),"wt")) == 0)
    STOPMSG("Cannot create difflist")
if ((fac = fopen(ACQUPATH("Indicator integral.txt"),"wt")) == 0)
    STOPMSG("Cannot create difflist")
    /*No go through each row in turn*/
TIMES(np)
{
RSR(v,wrpno)
RPROCNO(wrpno)
SETCURDATA
sprintf(intdir,"%s\\%s\\%i\\pdata\\%i\\integrals.txt",location,name,expno,procno);
/*Reference the spectrum.*/
/*If the reference peak is split, the program will reference based on the average shift*/
/*of the two peaks in the requested peak picking range which are within ppsens of the largest peak found*/
STOREPAR("f2p",f2pdss)
STOREPAR("f1p",f1pdss)
PP
 numPeaks = readPeakList(PROCPATH(0));
maxips=0.0;
    maxpsh=0.0;
    for (i=0; i<numPeaks; i++)
    {
                    peakIntensity = getPeakIntensity(i);
                    peakFreqHz = getPeakFreqHz(i);
                    peakFreqPPM = getPeakFreqPPM(i);
                    if (peakIntensity > maxips)
                    {
                        maxips = peakIntensity;
                        maxpsh = peakFreqHz;
                        maxpsp = peakFreqPPM;
                    }
    }
    /*Pick most downfield side of multiplet*/
    mintpp=maxips*ppsens;
maxpsp=0.0;
    for (i=0; i<numPeaks; i++)
    {
                                        peakIntensity = getPeakIntensity(i);
                                        if(peakIntensity>mintpp)
                                        {
                    peakFreqPPM = getPeakFreqPPM(i);
                    peakFreqHz = getPeakFreqHz(i);
                    if (peakFreqHz >= maxpsh)
                    {
                        maxpsp = peakFreqPPM;
                        maxpsh = peakFreqHz;
                    }
    }
                    }
/*Flips negative to choose most upfield peak of multiplet*/
for (i=0; i<numPeaks; i++)
    {
                                        peakIntensity = getPeakIntensity(i);
                                        if(peakIntensity>mintpp)
```

```
                                        {
                    peakFreqPPM = getPeakFreqPPM(i);
peakppmneg=peakFreqPPM*m;
maxpspneg=maxpsp*m;
                    if (peakppmneg >= maxpspneg)
                    {
                        minpsp = peakFreqPPM;
                    }
    }
                    }
    freePeakList();
/*References spectrum*/
ppmdif=maxpsp-minpsp;
cent=minpsp+ppmdif*0.5;
FETCHPAR("sf",&sf)
    sfn=sf+(cent-ref)*sf/(1e6);
    STOREPAR("sf",sfn)
/*Integrate spectra, read numbers and store integrals in text file*/
                    RMISC("intrng", text)
                    LI
fpnt=fopen(intdir, "r");
fgets(dummystr, sizeof(dummystr), fpnt);
while (fgets(dummystr, sizeof(dummystr), fpnt) != NULL)
{
                    /*Need to selectively elimate rows, then scan for numbers*/
if(linenum>=5)
{
                    (void) sscanf(dummystr,"%lf %lf %lf %lf",
                                                        &intnum,&ppmdn,&ppmup,&intgr);
                                                        /*DMSO is first, then indicator*/
                                                                            if(linenum==5)
                                                        {
                                                                            if(strcmp(ordans,"d")==0)
                                                                            {
                                                        fprintf(fref,"%f\n",intgr);
                                                                            }
                                                                                                if(strcmp(ordans,"u")==0)
                                                                            {
                                                        fprintf(fac,"%f\n",intgr);
                                                                            }
                                                        }
                                                                            if(linenum==6)
                                                        {
                                        if(strcmp(ordans,"d")==0)
                                                                            {
                                                        fprintf(fac,"%f\n",intgr);
                                                                            }
                                                                                                if(strcmp(ordans,"u")==0)
                                                                            {
                                                        fprintf(fref,"%f\n",intgr);
                                                                            }
                                                        }
                                                        intnum=0;
                                                        ppmdn=0;
                                                        ppmup=0;
                                                        intgr=0;
                                                        linenum++;
}
                    else
{
                                                        linenum++;
}
}
linenum=1;
fclose(fpnt);
                    /*Save spectra in procnos if requested (will overwrite!!)*/
                    if(strcmp(savans,"y")==0)
{
if(v==sav1)
{
WRP(sav1)
}
if(v==sav2)
{
WRP(sav2)
}
if(v==sav3)
{
WRP(sav3)
}
}
v++;
RPROCNO(phpno)
SETCURDATA
}
END
fclose(fac);
fclose(fref);
QUIT
```
